# Tropical bee species abundance differs within a narrow elevational gradient

**DOI:** 10.1038/s41598-021-02727-9

**Published:** 2021-12-03

**Authors:** Kristin M. Conrad, Valerie E. Peters, Sandra M. Rehan

**Affiliations:** 1 Department of Biological Sciences, Eastern Kentucky University, Richmond, KY USA; 2 Department of Biology, York University, Toronto, ON Canada

**Keywords:** Agroecology, Climate-change ecology, Community ecology

## Abstract

Insect pollination is among the most essential ecosystem services for humanity. Globally, bees are the most effective pollinators, and tropical bees are also important for maintaining tropical biodiversity. Despite their invaluable pollination service, basic distributional patterns of tropical bees along elevation gradients are globally scarce. Here, we surveyed bees at 100 m elevation intervals from 800 to 1100 m elevation in Costa Rica to test if bee abundance, community composition and crop visitor assemblages differed by elevation. We found that 18 of 24 bee species spanning three tribes that represented the most abundantly collected bee species showed abundance differences by elevation, even within this narrow elevational gradient. Bee assemblages at the two crop species tested, avocado and squash, showed community dissimilarity between high and low elevations, and elevation was a significant factor in explaining bee community composition along the gradient. Stingless bees (Tribe Meliponini) were important visitors to both crop species, but there was a more diverse assemblage of bees visiting avocado compared to squash. Our findings suggest that successful conservation of tropical montane bee communities and pollination services will require knowledge of which elevations support the highest numbers of each species, rather than species full altitudinal ranges.

## Introduction

A central theme in pollination ecology and pollinator conservation research asks how bee richness and abundance differs across land use types and along land use gradients^1^, as well as the implications of these differences for pollination services^2^. In the mountainous areas of the tropics it is equally important to know how bee richness and abundance varies with elevation for several reasons. First, the ability to predict bee response to climate change or other global change threats in tropical mountains depends upon knowledge of their altitudinal distribution, as well as abundance patterns along each species’ altitundinal range. For example, some species show lower abundances at range boundaries because biotic interactions, such as competition, predation, and the absence of mutualists, can further restrict ranges beyond the species’ fundamental niche^3^. A species occupying a broad altitudinal range, but that is only highly abundant within a narrow portion of that altitudinal range, should be as vulnerable to climate change as a species occupying a narrow altitudinal range. Second, understanding the abundance patterns of bees in tropical mountains also has many direct applications for tropical plant species. For example, for predicting the likelihood of loss of spatial/temporal co-occurrence from climate change or other global change impacts^2,4^. Finally, quantifying bee abundance along elevational gradients in tropical mountains has implications for humanity, as many economically important crops are grown in tropical mountains, and the abundance of bee species, specifically those documented as being important crop pollinators, can directly affect fruit quality and crop yield^5^.

Despite the invaluable service they provide, global distribution patterns of bees have only recently been quantified^6^. Global hotspots of bee diversity were found at mid-latitudes and arid environments, with lower bee species richness at lower latitudes^6^. This pattern is surprising given the increase in flowering plant species with decreasing latitude, as well as the increase in the proportion of flowering plant species relying on animal pollination at low latitudes. This result implies that tropical bees may have an even more critical role as pollinators because there are fewer tropical bee species yet many more flowering plant species dependent upon the services provided by tropical bees, and the specialization of these interactions compared to higher latitudes is still unresolved^7–9^. Tropical bee research has been hindered by a lack of taxonomists and taxonomic keys for some large tropical groups. For example, many genera of the Tribe Augochlorini, such as the diverse Augochlora spp., lack accurate species identification keys^10^. Owing to these limitations, bee conservation ecology in the tropics is lacking information on species richness, taxonomy, distribution, population dynamics and how climate change and other threats may impact topical bee species and bee pollination services^10^.

Since bees do not appear to follow typical global distribution patterns, their distribution along tropical mountains may also reveal unique patterns. Bee diversity and abundance are expected to decrease with increasing elevation, however, a mid-domain effect for tropical bees has not been tested because the lowest elevations evaluated were 870 m^11^ and 1800 m elevation^12^. The adverse weather conditions at high elevations in tropical mountains is posited to lead to reduced visitation rates of bees to plants, and a shift toward vertebrate pollination systems or buzz-pollination systems at high elevations^13,14^. Hence, with only a handful of studies we are still lacking a complete understanding of bee abundance patterns along tropical mountains, especially at mid-elevations where weather patterns are less severe, and in seasonally-arid systems which may be more favorable for bees.

Quantifying bee abundance patterns at mid-elevations of the seasonally-arid Pacific slopes of tropical America is also urgent since ectothermic mutualists in this region are already identified to be among the most at-risk species worldwide. First, all mutualists, which would include bees, are expected to have a higher risk of extinction from climate change and other global change impacts due to loss of mutualist partners^15^. In addition, owing to their narrow thermal tolerance, tropical ecototherms have been documented as a high-risk group to warming temperatures with substantial risk of extinction for tropical insects, specifically^16^. The upslope and poleward movement of species globally will lead to the creation of novel biotic interactions, communities, habitats, and moisture regimes, or may decouple important biotic interactions, likely posing a greater risk for tropical species^17^. Finally, the pacific slopes of Mesoamerica are expected to harbor the most at-risk species due to the combined effects of warming temperatures and altered precipitation regimes, including more frequent and severe droughts^18^. Thus, bees of the Pacific slopes of Central America are among the most at-risk taxa globally, facing many threats from climate change, including the decoupling of interaction partners, widowhood, desiccation, and shifting ranges upslope to novel temperature or precipitation regimes and novel communities^15–18^.

Here, we surveyed wild bee communities at 100 m elevation intervals along three spatially independent, replicate elevational gradients that include two life zones; a lower zone consisting of tropical dry forest (DF; 750–949 m elevation), and an upper zone consisting of tropical premontane forest (PMF; 950–1150 m elevation; see Supplementary Fig. S1 online) within the seasonally dry Northern Pacific Slope of Costa Rica. Smaller elevational gradients in the tropics have been studied less often because the differences are assumed to be marginal, however, plant-animal interactions can drastically vary over short distances on tropical mountains^17^. Studies along full elevational gradients can show the range extent of species but typically lack the resolution to detect whether species are equally abundant throughout their range. We used the replicate elevational gradients to ask: (1) if bee abundance and community composition differ by elevation, (2) what seasonal changes (wet season vs. dry season) in abundance and community composition are observed, and (3) how bee community composition of two economically important crop species changes along the elevational gradient and between the crops. To answer these questions, we focused on the western honey bee (*Apis mellifera*) and 29 native bee species from the two bee tribes, Ceratinini and Meliponini. We focused on these species for several reasons. First, the western honey bee and species of the two tribes comprised 75% of all bees collected (ca. 18,000 bees). Second, both tribes are more diverse in tropical compared to temperate regions, and bees of the tribe Meliponini are especially important in the tropics for their wax and honey production as well as their role in pollination^19^. Finally, we predicted that 1000–1100 m would be an important threshold for most Meliponine and Ceratinine bee species based on previous field observations in the study area^18^, and that, in contrast, the abundance of the western honey bee would be similar across elevations due to their generality in resource use and broad geographic range^20^. We also present the abundances of the 30 bee species collected from an additional elevation (0 m elevation) and forest type (Tropical Wet Forest) in Southwestern Costa Rica as supporting information for the bee species full altitudinal ranges.

## Results

A total of 13,524 bees representing 30 species from the tribes Ceratinini (5880 individuals), Meliponini (6291 individuals) and Apini (1353 individuals) were collected within 750–1150 m elevation (Supplementary Table S1). Sixteen of these 30 species were also collected from the 0 m elevation site (Supplementary Table S1).

Of the individuals collected from 750 to 1150 m elevation, a total of 5444 were collected in pan traps, 42 from vane traps, 1438 from aerial netting at flowers, 3723 from honey sprayed vegetation samples, and 2877 from 30-min observations at select plant species (Supplementary Table S2). Six of 30 species were represented by ≤ 11 individuals from all collection methods combined and three of the six were represented by only a single individual collected. The effect of elevation for these six species could not be statistically tested (Table 1; Supplementary Table S1). Sixteen of the remaining 24 bee species were Meliponine bees, seven were Ceratinine bees and one was the Western honey bee.

**Table 1:** GLMM and LMM results of the effect of elevation on each bee species’ abundance and presence in the various collection methods.

Bee Species	pan/vane trap + aerial net (abundance data)	honey spray (presence/absence)	30 min timed (presence/absence)
*Apis mellifera* Linneaus, 1758	NS (266)	Not tested (659)	NS (428)
**Ceratinine bees**			
***Ceratina auriviridis H. S. Smith, 1907***	Not tested (2)	Absent/not collected (0)	Not tested (3)
*Ceratina buscki* Cockerell, 1919	Not tested (17)	Not tested (3)	** (P < 0.05) (28)
***Ceratina chloris Fabricius, 1804***	Absent/not collected (0)	Absent/not collected (0)	Not tested (1)
*Ceratina cobaltina* Cresson, 1878	Not tested (10)	Absent/not collected (0)	NS (15)
*Ceratina dimidiata* Friese, 1910	Not tested (9)	Absent/not collected (0)	Not tested (9)
*Ceratina eximia* Smith, 1862	** (P < 0.05) (41)	Not tested (2)	NS (35)
*Ceratina ignara* Cresson, 1878	NS (24)	Not tested (3)	Not tested (2)
*Ceratina rectangulifera* Schwarz and Michener, 1954	** (P < 0.05) (4746)	Not tested (42)	** (P < 0.05) (160)
*Ceratina trimaculata* Friese, 1917	** (P < 0.05) (715)	Not tested (2)	** (P < 0.05) (10)
***Ceratina zeteki Cockerell, 1934***	Not tested (1)	Absent/not collected (0)	Absent/not collected (0)
**Meliponine bees**			
*Cephalotrigona zexmeniae* Cockerell, 1912	Not tested (3)	Absent/not collected (0)	** (P < 0.05) (58)
***Lestrimelitta mourei F. F. Oliveira and Marchi, 2005***	Not tested (1)	Absent/not collected (0)	Absent/not collected (0)
***Melipona beecheii Bennett, 1831***	Not tested (3)	Not tested (3)	Not tested (1)
*Melipona fallax* Camargo and Pedro, 2008	Not tested (10)	Not tested (2)	** (P < 0.05) (25)
*Melipona costaricensis* Cockerell, 1919	** (P < 0.05) (22)	Not tested (2)	Not tested (8)
*Nannotrigona mellaria* Smith, 1862	Not tested (5)	Not Tested (6)	** (P < 0.05) (26)
***Oxytrigona isthmina Gonzalez and Roubik, 2008***	Not tested (11)	Absent/not collected (0)	Absent/not collected (0)
*Partamona orizabaensis* Strand, 1919	* (P < 0.10) (150)	NS (592)	NS (234)
*Plebeia frontalis* Friese, 1911	** (P < 0.05) (34)	** (P < 0.05) (182)	** (P < 0.05) (48)
*Plebeia pulchra* Ayala, 1999	NS (94)	NS (412)	** (P < 0.05) (94)
*Scaptotrigona mexicana* Guérin-Méneville, 1845	NS (75)	NS (440)	NS (53)
*Scaptotrigona pectoralis* Dalla Torre, 1896	Absent/not collected (0)	Not tested (19)	Not tested (1)
*Tetragona dorsalis* Smith, 1854	** (P < 0.05) (27)	** (P < 0.05) (19)	** (P < 0.05) (118)
*Tetragonisca angustula* Latreille, 1811	NS (121)	** (P < 0.05) (547)	** (P < 0.05) (201)
*Trigona corvina* Cockerell, 1913	NS (113)	NS (576)	** (P < 0.05) (239)
*Trigona fulviventris* Guérin-Méneville, 1845	* (P < 0.10) (318)	** (P < 0.05) (66)	** (P < 0.05) (948)
*Trigona fuscipennis* Friese, 1900	** (P < 0.05) (39)	Not tested (10)	** (P < 0.05) (23)
*Trigona silvestriana* Vachal, 1908	** (P < 0.05) (35)	** (P < 0.05) (48)	** (P < 0.05) (84)
*Trigonisca buyssoni* Friese, 1902	NS (32)	NS (88)	** (P < 0.05) (25)

Bolded Italics species are the six species that could not be statistically tested from any collection method, with ≦11 individuals collected from all methods combined. NS is abbreviation for Not Statistically Significant. The abundance for each species is shown in parenthesis following the result of the statistical test.

From the combined samples collected from the replicate elevational gradients, including pan traps, vane traps, aerial netting and honey sprayed vegetation samples, a total of 25 bee species were collected from 800 m elevation, 24 species from 900 m elevation, 22 species from 1000 m elevation and 21 species from 1100 m elevation (Supplementary Fig. S1). The Chao species richness estimator estimated species richness of the focal bee taxa at 800 m elevation to be 33.12 ± 8.92 species, 27.00 ± 3.35 species at 900 m elevation, 24.72 ± 2.86 species at 1000 m elevation, and 23.33 ± 2.49 species at 1100 m elevation.

### Bee abundance from pan, vane traps, and aerial netting

**Figure 1:**
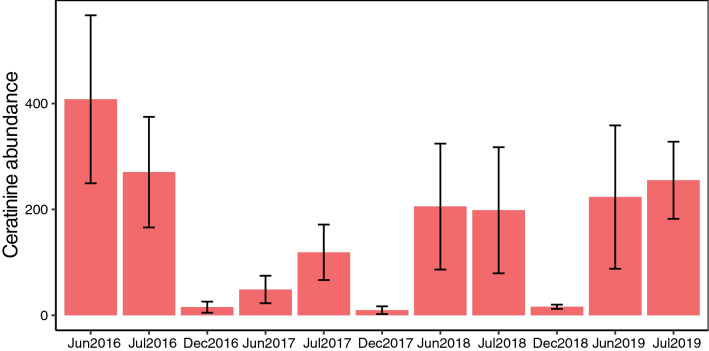
Mean (± SE) abundance of Ceratinine bee species, *C. rectangulifera* and *C. trimaculata* collected from pan traps, vane traps, and aerial netting at vegetation across three replicate elevational gradients, comparing wet season (June/July) and dry season (December).

Twenty-eight of the 30 bee species were collected from pan traps, vane traps and aerial netting methods combined. Five of the 28 species were included in the species that could not be statistically tested because they were those represented by ≤ 11 individuals (Table 1; Supplementary Table S1). After removing these five species, a further six species were represented by ≤ 17 individuals in pan traps, vane traps and aerial net samples, and these species could not be statistically tested from these collection methods. Of the remaining 17 species, the abundance of ten species differed by elevation, while the abundance of seven species did not differ by elevation (Table 1, Supplementary Fig. S2). The two most abundant species collected by these methods combined were *Ceratina rectangulifera* and *Ceratina trimaculata*, followed by *Trigona fulviventris* and *Apis mellifera* (Table 1). Season of collection had a significant effect on *C. rectangulifera* and *C. trimaculata* abundance (i.e., the two species combined; *X*^2^_2_ = 18.271; *p* < 0.001). On average, fewer individuals were collected during the dry season (14 ± 3.97 s.e.m.) compared to the wet season (216 ± 37.75 s.e.m.; Fig. 1). *A. mellifera* abundance was not significantly different by season (*X*^2^_*2*_ = 1.434; *p* = 0.49). *T. fulviventris* abundance was significantly different by season (*X*^2^_*2*_ = 56.062; *p* =  < 0.001), with more individuals collected during the dry season compared to the wet season. For the overall bee community, season significantly influenced bee community composition (ANOSIM R = 0.5507, p = 0.003; Supplementary Fig. S3). Ceratinine bees tended to be observed more frequently in the wet season while Meliponine bees tended to be observed more frequently in the dry season (Supplementary Fig. S4). Temporal changes in the bee community was high (0.729 sorenson) and mainly characterized by turnover (0.573) compared to nestedness (0.156).

### Meliponine bee presence/absence from honey sprayed vegetation

Although *Apis mellifera* and five Ceratinine bee species were collected at honey baits, only Meliponine bee species were statistically tested using data from this collection method. Sixteen Meliponine bee species were collected from honey sprayed vegetation. Of these, six species were observed in < 5 of 116 samples and could not be statistically tested from this collection method (Table 1; Supplementary Table S4). Infrequently collected bees included *M. beecheii* and *M. fallax,* which were more frequently observed in the upper elevation zone, > 950 m elevation, and *S. pectoralis*, *M. costaricensis* and *T. fuscipennis,* which were more frequently observed in the lower elevation zone, < 950 m elevation. One infrequently collected bee, *N. mellaria*, was only observed in the lower elevation zone, < 950 m elevation. The presence of five species differed by elevation, while the presence of five other species did not differ by elevation (Table 1; Supplementary Table S4).

### Bee presence/absence from timed samples at flowering plant species

**Figure 2:**
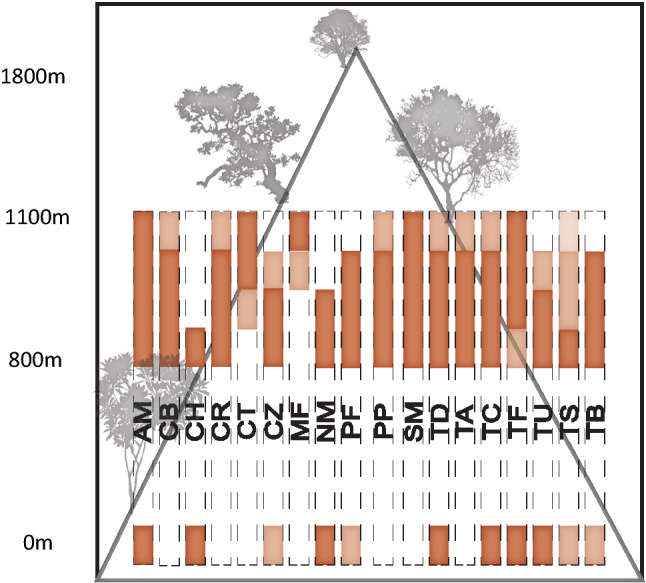
Abundance by elevation of 18 bee species collected in Northwestern Costa Rica. Darker brown indicates a statistically higher abundance for that bee species, with each elevation sampled comprising one-fourth of the bar from 800-1100 m elevation. Bee abundance differences were not statistically assessed between 800 and 0 m elevation since collection methods differed. Dark brown at 0 m elevation indicates a similar or higher abundance to 800 m, lighter brown indicates a lower abundance compared to 800 m. White blocks for an elevation band indicate that no individuals were collected for that bee species at that elevation. *Apis mellifera* (AM) and *Scaptotrigona mexicana* (SM) are included as bee species that did not statistically differ in abundance by elevation, and *Ceratina chloris* (CH) is included as one species that was not collected in sufficient numbers to statistically test, but was found in high numbers at 0 m elevation and only collected from 800 m elevation in the mountains.

Twenty-seven of the 30 bee species were collected from timed samples at flowering plant species. Three of the 27 species were included in the species that could not be statistically tested because they were those represented by ≤ 11 individuals (Table1; Supplementary Table S1). After removing these three species, an additional four species were represented by ≤ 10 individuals in timed samples, and these species could not be statistically tested from this collection method. Of the remaining 20 species, the presence of 15 bee species differed by elevation, while the presence of five bee species was not significantly different by elevation (Table 1, Supplementary Fig. S5, Supplementary Table S5). Twelve of the 15 bee species with significant differences by elevation were more abundant at lower elevations, and three of the 15 bee species were more abundant at higher elevations (Fig. 2).

### Bee community composition by elevation

**Figure 3:**
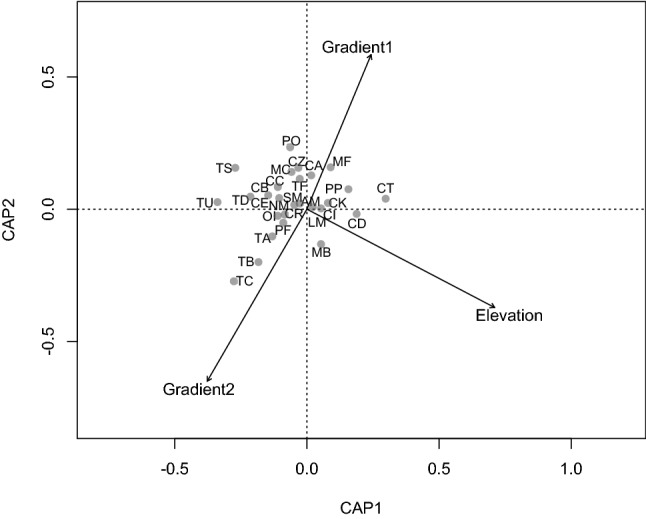
Bee species relative abundance by elevation and elevational gradient ID. Elevation was significant in explaining ~ 23% of the variation in bee community composition.

A total of 6866 bees belonging to 28 species were used in the constrained ordination. The factor of interest, elevation, explained 23% of the variation in bee community composition (*F*_pseudo1,8_ = 2.63, *p* = 0.012; Fig. 3). The blocking factor, elevational gradient ID, was not statistically significant (*F*_pseudo2,8_ = 1.22, *p* = 0.260). Examples of bee species associated with higher elevations included *C. trimaculata, P. pulchra, M. beecheii*, *M. fallax* and *C. dimidiata*. Examples of bee species associated with lower elevations included *C. rectangulifera*, *T. silvestriana, P. frontalis*, *C. eximia, M. costaricensis, N. mellaria, T. fuscipennis, T. dorsalis, T. corvina, T. buyssoni* and *T. angustula*. The permutational MANOVA showed an effect of elevation on species abundances (*F*_pseudo3,8_ = 760.5, *p* = 0.011) but no significant effect of the blocking factor, elevational gradient ID (*F*_pseudo2,8_ = 43.4, *p* = 0.276).

### Bee visits to crops and community similarity

**Figure 4:**
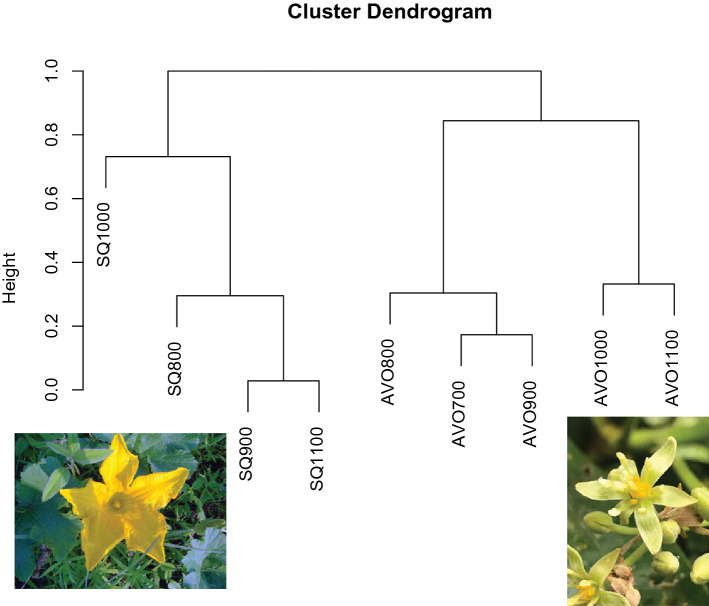
Cluster dendrogram for bee community visiting squash and avocado flowers. Chao dissimilarity index was used to quantify bee community dissimilarity.

A total of 145 individuals representing 21 species were collected from *Persea americana* flowers (Supplementary Table S6). A total of 223 individuals representing 9 species were collected from *Cucurbita pepo* flowers (Supplementary Table S6). Species accumulation curves for the two crops indicate that sampling effort for squash was adequate, while the curve for avocado did not appear to approach an asymptote (Supplementary Fig. S8). The bee community visiting avocado was more species rich than squash (Supplementary Fig. S9), and hosted a distinct assemblage of bee species, sharing only 4 of 26 species collected (Fig. 4; Supplementary Table S6). For avocado, the lower elevations 700, 800, and 900 m elevation shared more similar communities, while bee communities of 1000 and 1100 m elevation were similar. For squash, the bee community at 1000 m elevation was more distinct from the other elevations (Fig. 4). Crop species explained 57% of the variation in bee community composition (*F*_pseudo1,6_ = 12.75, *p* = 0.001) while elevation explained 17% of the variation in bee community composition (*F*_pseudo1,6_ = 3.93, *p* = 0.029; Supplementary Fig. S10). Threshold species identified as species with abundances that decreased with increasing elevation included *Trigona corvina* and *Tetragonisca angustula* and species that increased in abundance with increasing elevation included *Partamona orizabaensis*, *Trigona fulviventris* and *Melittoma* sp.1. No other species met the criteria for purity and reliability in the analysis.

## Discussion

Studies within the last two decades have repeatedly found that tropical ectotherms, including tropical insects, tend to have narrow thermal tolerances, making them an especially vulnerable group to global temperature rise^17^. Our study contributes to this body of knowledge by taking a statistical approach to quantify abundance patterns within a narrow elevational gradient to ask if the factor of elevation, specifically, explains variation in abundance patterns. Abundance differences along the elevational gradient revealed which bee species were more frequently observed in the lower portions of the gradient studied and which bee species were more frequently observed in the upper portions of the gradient studied.

Our results support the arguments of HilleRisLambers et al.^3^, that more work is needed to explore the relative contributions of biotic interactions and climatic factors in shaping species distributions and quantifying their responses to climate change, since biotic or abiotic factors could be responsible for the observed patterns of lower abundance at the upper or lower range limits of the species we studied^21^. For example, the mariola bee (*Tetragonisca angustula*) is a small-bodied (< 6 mm), economically-important stingless bee that is often kept for its medicinal honey^19^. One farmer in the study area reported having attempts to keep mariola hives at 1140 m elevation fail due to consistently low temperatures (personal communication). In the study area, mariola hives have not been found above 1000 m, and our results display a significantly lower abundance of this species from 1000 m elevation to 1100 m elevation. In a study of Meliponine bee altitudinal distributions along a single, but different elevational gradient nearby, no *T. angustula* were collected above 1000 m elevation^22^. Another example, however, highlights the role of biotic interactions in shaping abundance patterns. The two small carpenter bee species most commonly collected in pan traps were found to occur at all elevations, however, *C*. *rectangulifera* was observed more frequently in the lower zone, while *C. trimaculata* was observed more frequently in the upper zone. *Ceratina rectangulifera* and *trimaculata* are nearly morphologically identical species, with similar body size and color^23,24^, and no differences were found in flowering plant species associations or nesting habitat. Therefore, owing to similarity in resource use, these two bee species may specialize in elevation to avoid competition^25^.

Bees, like many organisms, can be sensitive to landscape context. We aimed to reduce the confounding effects of land use by conducting our study in an area of the tropical countryside with a similar landscape context along the elevational gradients and throughout the valley studied, as well as statistically, by replicating smallholder farms and sampling paths at each 100 m elevation studied. Despite the landscape differences among the three elevational gradients, an effect of elevation was still statistically detected in the abundances of many bee species occurring along the elevational gradients, and elevation explained 23% and 17% of the variation in bee community composition, and bee assemblage of two crop species, respectively. Finally, our statistical models accounted for the variation introduced by location (i.e. elevational gradient ID) in order to focus on the factor of interest, elevation^26^. In addition to land use, a factor commonly acknowledged as having a substantial influence on insect abundance, these results highlight the important role of elevation in explaining the relative abundance of bee patterns in tropical mountains, and indicate that elevation on tropical mountains, even within a narrow, 400 m elevation difference, is an important factor shaping bee assemblages.

Our crop visitation data shows low similarity in the community of bees that visit crop flowers between crop species and also between some elevations, indicating the importance of elevation in determining the pollinator assemblages occurring at each crop species, as well as crop pollination effectiveness^27^. When bee species in the study area shift their ranges upslope, as climate change research predicts^28^, pollination services for economically important crop species may become unreliable, owing to spatial mismatches^29^. We did not evaluate the effectiveness of each bee species for fruit set or fruit quality in our study, however, our results suggest that, as a result of warming temperatures, novel bee communities are likely to be formed in tropical montane landscapes in the future, with an unknown impact to pollination services^30^. Our data shows a unique bee community visiting each of the two crops studied, highlighting that crop species in the tropical mountains likely rely on more specialized assemblages of bees. A recent review of avocado pollination found that stingless bees are as effective as the honey bee at avocado pollination and suggested that the contribution from wild pollinators to avocado pollination may be higher in Central American countries, owing to the co-evolution of pollinators and avocados in Central America, but found that no studies of avocado pollination had yet been conducted in any Central American country^31^. Our study found that 10 of 19 bee species collected from avocado flowers were stingless bee species, and for 7 of those 10 stingless bee species, we observed abundance differences by elevation; all 7 stingless bee species were more frequently observed in the lower elevation zone. Vulnerability to climate change has already been shown for a number of crop species throughout the Neotropics^32^, and our study, which is the first to quantify tropical bee abundance patterns across an elevation gradient, supports that pollination services in tropical montane landscapes may be highly vulnerable to warming temperatures. In our study, *Apis mellifera* abundance did not vary with elevation. Since *A. mellifera* is an effective pollinator, this result may demonstrate the resiliency of pollination services in the tropics. However, if wild honey bee populations decline, then local farmers will have to rely more heavily on native species, particularly the highly abundant social stingless bees (Tribe Meliponini). Wild bee species richness, total bee abundance, functional group diversity, and the abundance of the most effective bee species have all been linked to crop yield, fruit quality and fruit size, however, these relationships have yet to be verified for most tropical crop species^5,31,33,34^. This highlights an urgent need to protect multiple metrics of bee populations, including relative abundances.

Decreasing predictability in rainfall patterns and severe drought are also expected to severely impact species of the Pacific slopes of Mesoamerica^18^. Precipitation data combined with long-term data of annual and seasonal fluctuations in insect abundances could reveal which species may be more sensitive to moisture and precipitation. For example, while our data is not of a sufficient timespan to statistically test and draw conclusions^35^, we observed that the total abundance of the two most commonly collected Ceratinine species from pan traps was much higher during the rainy season compared to the dry season (Fig. 1). Additionally, we observed variation in the annual abundance of the two Ceratinine species collected in pan traps across the three replicate elevational gradients. Comparing rainfall during the onset of the rainy season (1April- 1June) using a weather station located at the University of Georgia Costa Rica campus (1100 m elevation), we found a normal amount of rainfall recorded (462 mm rainfall over 35 days) in 2016, while a severe drought was recorded in 2017 (< 18 mm rainfall over 14 days), and a severely delayed rainy season was observed in 2018 (242 mm rainfall over 9 days beginning 23 May). Using the elevational gradients as replicates, mean abundance of the two Ceratinine species was 408 ± 158 s.e.m. individuals in June 2016, 48 ± 25 s.e.m. individuals in June 2017, and 201 ± 119 s.e.m. individuals in June 2018, suggesting that drought or delay in the onset of the rainy season could influence the population size of some bee species in the seasonally dry tropical mountains. While other potential explanations exist for these patterns, moisture is one of the most important abiotic factors affecting the life of terrestrial organisms, and future work should aim to quantify desiccation tolerance in bee species, especially bee species of seasonally-arid montane environments, as these systems are predicted to experience the most drastic changes in precipitation regimes^18^.

Studies from temperate montane pollinator communities have predicted that species will expand their ranges at the cool, upper elevational limits and contract their ranges at the warm, lower elevational limits, creating novel communities where competition may lead to local extirpation or extinction^28^. Most research aimed at quantifying altitudinal distributions of species in Costa Rica has been conducted on the two mountain ranges in the southern half of the country, which have much taller peaks (> 2500 m elevation) compared to the two mountain ranges in the northern half of the country, the Guanacaste and Tilarán Cordilleras, that are comprised of relatively short mountains with the highest peaks from 1500 to 2000 m elevation^36^. For example, the Monteverde mountain, where this study was conducted, peaks at 1800 m. In tropical mountains, such as the Monteverde mountain, ecologists have already observed the upslope movements of species in response to warming temperatures^2^ and further upslope movements of 600 m are predicted by the end of the century^28^. With upslope movements of 600 m, the lower zone specialists in our study (800–1000 m elevation) will replace or face competition from species at the top of the mountain (1400–1600 m elevation), while upper zone specialists (1100 m elevation) are species that will be confronted with the disappearance of their thermal habitat on these short mountains. Several of the upper zone specialists are large bodied *Melipona* spp. which are economically important stingless bee species that were traditionally kept throughout Central America for honey, and are now considered to be rare species throughout much of their previous range^19^. Our results highlight that there is an urgent need to experimentally determine the mechanisms responsibe for the observed abundance patterns as outlined in HillleRisLambers et al.^3^, to increase understanding of the factors shaping bee distributional patterns on tropical mountains, as well as how food security in local communities may be impacted by the future formation of novel bee communities.

Our study spanned multiple years, with a total of 262 h collecting bees directly from flowers, as well as 44 days of pan and vane traps, and netting bees from roadside, farm and honey sprayed vegetation. After extensive sampling (> 13,000 bees from the focal tribes and > 18,000 bees total), eight bee species of the 30 collected in the study area from the focal tribes were collected too infrequently to statistically analyze differences in their abundance across the elevation gradient, suggesting that these species could be rare in the study area. Other explanations may also account for these low numbers, such as that we under sampled species with peak abundances occurring above or below the study area or did not sample sufficiently from these bee species’ preferred plant species. For example, *C. chloris* was collected only once across the elevation gradient at 800 m, but 281 individuals were collected at the 0 m elevation site. However, if these bee species are rare in the study area due to land use change, loss of preferred floral resources, or have naturally occurring low local population sizes, then these species may be at greater risk of climate change impacts^37^. In addition to these rare bee species, 19 bee species in our study were ≤ 6 mm, and small-bodied bee species are also predicted to have an increased extinction risk compared to larger bees^38^. Our results, which revealed that tropical bees show abundance differences over small elevation differences, as well as differences by elevation in community composition at crop flowers, combined with these additional risk factors, highlight the urgent need for more work focused on understanding tropical bee distributional patterns as well as work aimed at developing conservation and mitigation recommendations to support healthy populations of mountain-dwelling tropical bee species.

In addition to the bee species that were collected too infrequently to statistically analyze, some bee species were found to not statistically differ by elevation in the flower visitation data, but were shown to be associated with lower elevations in the ordination (e.g. *C. eximia* and *C. cobalitina*). Collection numbers from all methods combined for *C. eximia* show that this species was collected at a relatively consistent frequency at all elevations except 1100 m; only 2 individuals were collected from 1100 m elevation while 28, 25 and 23 individuals were collected from 800 m, 900 m and 1000 m elevations, respectively (Supplementary Table S1).

In summation, focusing on a subset of bee species from the Pacific slopes of Costa Rica, we report that the abundance of 18 of 24 bee species were statistically different by elevation, and 2 of the remaining 6 species were collected too infrequently to statistically estimate differences by elevation. Over short distances on tropical mountains, our study found variation in the abundance of 14 of 16 bee species belonging to the tribe Meliponini, an economically important group of pollinators for tropical trees and crops^2,39^, and in the abundance of 4 of 7 bee species belonging to the tribe Ceratinini. Ceratinine species are also likely to be important pollinators^40^, however, few studies regarding Ceratinine bee pollination services have taken place in tropics, and the topic is only recently being explored in the U.S.^41^.

## Methods

### Study site

We established three spatially independent, replicate elevational gradients in the San Luis Valley of Monteverde, Puntarenas, Costa Rica (10°16′ N, 84°48–49′ W) in 2012 (Supplementary Fig. S6), with the goal of quantifying bee distibution and abundance patterns, detecting range shifts, and developing a baseline for quantifying local population status over time (trajectories). Each of the elevational gradients follows along a dirt and gravel roadside, and are separated by 1 km, with the lowest elevation of each gradient at approximately 750–800 m a.s.l., and the highest at approximately 1075–1150 m a.s.l. Along each elevational gradient, sampling paths were located at 800 m, 900 m, 1000 m and 1100 m elevation, for a total of 12 sampling paths.

The San Luis Valley is located on the Pacific slope of the Tilarán mountain range and is comprised of tropical dry forest with low humidity during the dry season, and pre-montane forest with consistently humid conditions^42^. The San Luis Valley is typical of the tropical countryside, consisting of smallholder farms and substantial forest remnants scattered throughout, creating a complex of mixed landcover that is consistent across the gradient. In San Luis, there are distinct rainy and dry seasons, with the rainy season occuring May–November (mean monthly precipitation: 200–600 mm) and the dry season occuring December–April (mean monthly precipitation: 50–150 mm)^43^.

Bee abundance data from 0 m elevation was collected during a previous study conducted from June to July 2017–2019 and December 2017 in the Osa Peninsula (OP) of Costa Rica^44^. The life zone of the OP corresponds to tropical wet forest. Bees were collected from ornamental and naturally occurring flowering plant species in eight sampling locations that included roadside, home gardens and smallholder farms, all located at 0 m elevation.

### Focal bees

Species in the tribe Ceratinini are highly diverse and globally distributed, with 350 species worldwide, and 15 species in Costa Rica^45^. Michener^23^ recognized one genus, *Ceratina*, within this tribe, with 21 subgenera. Individuals in this tribe range from 2.2 to 12.5 mm in length. The bee tribe Meliponini consists of several hundred described species arranged into 21 genera^23^. Fifty species are known to occur in Costa Rica^19^, however, this is likely an underestimate^46^. Meliponine bees are the primary visitors of many flowering plants in the tropics, are well established as important crop pollinators throughout the tropics^2^ and range from 1.8 to 13.5 mm in length. *Apis mellifera*, the western honey bee, is found throughout South and Central America and southern portions of the United States^47^. *Apis mellifera* is considered a supergeneralist foraging species and has been documented as the most abundant pollinator visitor to various plant taxa^48^. No known residential apiaries or beekeeping farms exist in the study area.

### Focal crops

*Persea americana* Mill. is native to Mexico, Central and South America, however, it is widely and commercially cultivated in tropical and Mediterranean climates globally^49^. Avocado flowering behavior expresses a diurnally synchronous protogynous dichogamy; each flower opens twice, first as a female, then as a male during the second opening^50^. The two complementary flowering stages occur simultaneously on separate individuals and, under proper conditions, will flower at opposite stages in order to ensure cross-pollination^51^.

Most species in the genus *Cucurbita* are native to Mesoamerica^52^. *Cucurbita* species are annual vine or bush crops that are monecious, thus requiring biotic pollination for fruit set^53,54^. *Cucurbita* produces large, showy yellow-orange flowers that have a brief flowering period; flowers open before or by sunrise and close by the afternoon^53,54^.

### Bee sampling

Sampling for bees was conducted along each sampling path, located at 800, 900, 1000, and 1100 m elevation, during the months of June- July of 2016–2019, and December of 2016–2018. All sampling paths began at the elevation specified above, and followed upslope along the elevational gradient for 150 m. As all sampling paths followed along the roadside of the elevational gradients, all replicate elevations were > 1 km apart, with the three 800 m sampling paths being the closest spatially (Supplementary Fig. S6). The distance between elevations along the same elevational gradient varied based on the steepness of the slope, but the minimum distance was 300 m. A combination of collection methods were used. Pan traps were 30 plastic 2.5 oz cups painted florescent yellow, florescent blue and white, then filled 2/3 with soapy water and placed linearly, along each sampling path, approximately 5 m apart for a total length of 150 m. Vane traps included one yellow and one blue vane trap; each filled to approximately 1/6 with soapy water and hung from a tree branch located near the center of each sampling path. Pan and vane traps were placed out three times at each sampling path in June-July each year and one time at each sampling path in December each year. Pan and vane traps were placed out from 800 to 1400 h except during periods of heavy precipitation. Aerial nets and kill jars were also used to collect bees from all flowering plants along the 150 m sampling path. Aerial netting was conducted after traps were placed out at 800 h, and two observers walked slowly along each sampling path collecting all flower visitors for ca. 3 h. Finally, one large, leafy plant near the middle of the 150 m sampling path was sprayed with a 2:1 water: honey solution that was used to target Meliponini bees, as pan and vane traps do not effectively capture bees in this tribe^55^. Aerial netting and honey sprayed vegetation sampling were conducted three times at each sampling path in June-July each year and one time at each sampling path in December each year. Bees were also collected during 30-min observation periods at specific flowering plant species that occurred on small farms bordering the three replicate elevational gradients from 800 to 1400 h in June, July and December 2017–2019. From 2016 to 2019, a total of 193 pan trap, 86 vane trap, and 181 aerial net from flower samples were collected. From 2018–2019, a total of 116 aerial net from honey sprayed vegetation samples were collected. From 2017 to 2019, a total of 521 samples from 30-min observations at select flowering plant species were conducted.

Bees were collected from avocado and squash flowers located on 15 smallholder farms and residential properties ranging from 1 to 3 ha each. Farms were initially selected for this study by elevation to ensure that three to four farms were sampled at each of the four elevations, 800, 900, 1000, and 1100 m elevation, and by spatial location to ensure that farms were > 250 m apart. Farms were also selected based on the owner’s willingness to allow us to plant squash on their property and the presence of avocado trees on or near the property. Avocado trees were naturally occurring or planted for household consumption, while all squash seeds were planted directly in the ground on farms. While most farms had both squash and avocado available for sampling, on some farms we were unable to locate avocado trees or plant squash (Supplementary Fig. S6). Each smallholder farm and residential property had diverse non-crop herbaceous plants, ornamentals, fencerows, windbreaks, shade trees, and were embedded in a matrix of secondary forest patches and cattle pasture. All smallholder farms also differed in crop species included on the property, but most had areas of coffee, citrus, guava, mango, and some small vegetable gardens. Bee sampling from avocado and squash was conducted during the months of June, July and December of 2018–2019 from 800 to 1400 h daily. Any bees observed contacting the flowers reproductive parts during a 30-min observation period were collected using aerial nets and jars. A total of 47 observations were conducted on avocado trees, and a total of 32 observations were conducted on squash plants.

All collected bees were preserved and exported to Eastern Kentucky University for identification and processing. Meliponine bees were identified to species using identification keys^23,56^. A Ceratinine bee reference collection was used to identify all Ceratinine bees, and reference bees were identified by S. Rehan. Bees collected from avocado and squash that were not Ceratinine or Meliponine bees were either identified to species or genus and morphospecies.

### Environmental variables

To quantify the monthly average and maximum temperature differences between the lowest and highest elevations of the study area, we used historical average and maximum temperature data (1970–2000) from WorldClim version 2.1^57^, a high resolution (~ 1km^2^) global monthly climate dataset. Data was extracted and analyzed using the ‘raster’ and ‘maps’ packages of R versions 3.1–5 and 3.3.0^58,59^, and visualized using the ‘ggplot’ function in the ‘ggplot2’ package of R version 3.3.0^60^. The mean annual temperature difference is approximately 0.7 °C between 800 and 1100 m elevation (Supplementary Fig. S7).

### Data analysis

To determine if bee species abundance differs by elevation or season, generalized linear mixed models (GLMM) and linear mixed models (LMM) were constructed with bee species abundance or presence/absence as the response variable. Total bee abundance data for each species was obtained by combining all pan trap, vane trap, and aerial netting from flowers samples collected along each sampling path. Bee presence/absence data was used to model data obtained from honey spray vegetation samples (n = 116) and from 30-min observations at select flowering plant species (n = 521). We used bee presence/absence as the response variable for these two datasets because (1) it allowed us to include observations when each particular bee species was not observed, and (2) Meliponine bees are eusocial and therefore using total abundance data collected from attracting them to a bait is not recommended^61^. Bee abundance data were modeled using GLMMs or LMMs constructed for each species. Bee abundance was summed by year, and either log transformed to meet the assumptions of normality, or modeled with a negative binomial error distribution. All models included the fixed effects of either elevation or season and the random effect of the elevational gradient ID. Bee species collected in too few numbers to use the elevational gradient as a replicate (random effect) for assessing differences in abundance by season were summed across the elevational gradients and the summed abundance per sampling period was used in a GLM or LM as the response variable. Bee presence/absence data was modeled using GLMMs constructed for each species with a binomial error distribution. All models for honey bait samples included the fixed effect of elevation and the random effect of the elevational gradient ID. All models for 30-min observation samples included the fixed effect of elevation and the random effect of farm ID. A Chi-square goodness-of-fit test was used to test for distribution fit. Likelihood ratio tests were used to assess the significance of the fixed effects^26^. All LMM and GLMM models were conducted using the ‘lmer’ and ‘glmer’ functions in the ‘lme4’ package in R version 3.6.2^62^. A Tukey’s posthoc test in the ‘multcomp’ package was used to test which elevations were statistically different.

To evaluate how bee community composition differed by season and across the elevation gradient, we constructed one species by sampling period matrix (n = 15) and one species by sampling path (site) matrix (n = 12). Total bee species abundance data for each cell was summed from all pan trap, vane trap, and aerial netting samples for that column x row combination. To test the effect of season, we first constructed a dissimilarity matrix using the Chao dissimilarity index, which accounts for unseen species. Next we used the dissimilarity matrix to conduct a permutational analysis of similarity (ANOSIM) with 999 iterations. To partition the seasonal changes in bee community composition into turnover and nestedness components we converted the abundance matrix to a presence/absence matrix and used the ‘beta.multi’ function in the R package ‘betapart’^63^. To test the effect of elevation, we fitted a distance-based RDA using the Bray–Curtis distance index that was constrained by the factor of interest, elevation, and the blocking factor of elevational gradient ID. Bee abundances were quarter power transformed in order to reduce the effect of large variances in species abundances as some bee species were collected in the thousands, while fewer than ten individuals of other species were collected. We tested the significance of elevation and elevational gradient ID using a permutational test set at 999 permutations. The permutational analysis of variance provides a pseudo F-stat for each term in the model. The constrained ordination and significance test was conducted using the ‘vegan’ package of R version 2.5-6^64^ with the function ‘capscale’. In addition, a permutational MANOVA with 999 iterations was conducted using the ‘manylm’ function in the ’mvabund’ package of R^65^. We tested the relationship between spatial distance and bee community dissimilarity using the function ‘decay.model’ in the package ‘betapart’ and found no relationship (p = 0.85, Supplementary Fig. S11).

To determine the adequacy of sampling effort and to compare species richness of squash and avocado bee communities, species accumulation curves and rarefaction curves were constructed using the ‘accumcomp’ function in the BiodiversityR package of R version 2.11-3^66^. Rarefaction curves were re-scaled by abundance on the x-axis and include 95% CI. To quantify bee community similarity between crop species and elevations, we first constructed a site x species matrix of bee abundances and calculated a community dissimilarity matrix using the Chao dissimilarity matrix. We then constructed a cluster dendrogram using the ‘hclust’ function in the ‘vegan’ package of R. To test the effect of elevation and crop species in the bee communities visiting the two crop species, we fitted a distance-based RDA using the Bray–Curtis distance index. Bee abundances were quarter power transformed. We tested the significance of elevation and crop species using a permutational test set at 999 permutations. The constrained ordination and significance test was conducted using the ‘vegan’ package of R.

## Supplementary Information

Supplementary Information.

## Data Availability

The data that support the findings of this study are openly available in Dryad Digital Repository at http://doi.org/[doi], reference number [reference number].
