# Supplementary material for: Tropical bee species abundance differs within a narrow elevational gradient
Source: Sci Rep. 2021 Dec 3;11:23368. doi: 10.1038/s41598-021-02727-9 (PMC8642410; doi:10.1038/s41598-021-02727-9)
Supplement: Supplementary file 1 — Supplementary Information. [file 41598_2021_2727_MOESM1_ESM.docx]

**Title:** Tropical bee species abundance differs within a narrow elevational gradient.

Author list and affiliations:

Conrad, Kristin M.^^[[1]](#footnote-1)^^*, Peters, Valerie E.^1^, Rehan, Sandra M.

^1^Department of Biological Sciences, Eastern Kentucky University, Richmond, KY, USA

*Corresponding author: kristin.conrad1230@gmail.com

**APPENDIX S1-**Supplementary tables

Supplementary Table S1. Abundance of bee species collected near each elevation in the San Luis Valley (800m-1100m elevation) and the Osa Peninsula (0m elevation), Puntarenas province, from 2016-2019.

| Bee Species (Abbreviation) | 0 m | 800 m | 900 m | 1000 m | 1100 m |
| --- | --- | --- | --- | --- | --- |
| *Apis mellifera* Linneaus, 1758 (AM) | 545 | 393 | 249 | 387 | 324 |
| *Cephalotrigona zexmeniae* Cockerell, 1912 (CZ) | 4 | 8 | 52 | 1 | 0 |
| *Ceratina auriviridis* H. S. Smith, 1907 (CA) | 0 | 3 | 0 | 2 | 0 |
| *Ceratina buscki* Cockerell, 1919 (CB) | 1 | 22 | 14 | 10 | 2 |
| *Ceratina chloris* Fabricius, 1804 (CH) | 281 | 1 | 0 | 0 | 0 |
| *Ceratina cobaltina* Cresson, 1878 (CC) | 0 | 8 | 7 | 7 | 3 |
| *Ceratina dimidiata* Friese, 1910 (CD) | 0 | 1 | 5 | 7 | 5 |
| *Ceratina eximia* Smith, 1862 (CE) | 0 | 28 | 25 | 23 | 2 |
| *Ceratina ignara* Cresson, 1878 (CI) | 0 | 8 | 5 | 6 | 10 |
| *Ceratina rectangulifera* Schwarz and Michener, 1954 (CR) | 0 | 1098 | 1827 | 1267 | 756 |
| *Ceratina trimaculata* Friese, 1917 (CT) | 1 | 44 | 152 | 219 | 312 |
| *Ceratina zeteki* Cockerell, 1934 (CK) | 0 | 0 | 0 | 0 | 1 |
| *Lestrimelitta mourei* F. F. Oliveira and Marchi, 2005 (LM) | 0 | 0 | 0 | 1 | 0 |
| *Melipona beecheii* Bennett, 1831 (MB) | 0 | 0 | 1 | 5 | 1 |
| *Melipona fallax* Camargo and Pedro, 2008 (MF) | 0 | 4 | 4 | 2 | 27 |
| *Melipona costaricensis* Cockerell, 1919 (MA) | 163 | 6 | 20 | 0 | 6 |
| *Nannotrigona mellaria* Smith, 1862 (NM) | 107 | 19 | 18 | 0 | 0 |
| *Oxytrigona isthmina* Gonzalez and Roubik, 2008 (OI) | 0 | 10 | 0 | 1 | 0 |
| *Partamona orizabaensis* Strand, 1919 (PO) | 198 | 296 | 154 | 86 | 440 |
| *Plebeia frontalis* Friese, 1911 (PF) | 5 | 22 | 160 | 76 | 6 |
| *Plebeia pulchra* Ayala, 1999 (PP) | 0 | 42 | 194 | 253 | 111 |
| *Scaptotrigona mexicana* Guérin-Méneville, 1845 (SM) | 0 | 26 | 372 | 64 | 106 |
| *Scaptotrigona pectoralis* Dalla Torre, 1896 (SP) | 7 | 2 | 17 | 0 | 1 |
| *Tetragona dorsalis* Smith, 1854 (TD) | 72 | 70 | 24 | 65 | 5 |
| *Tetragonisca angustula* Latreille, 1811 (TA) | 0 | 331 | 282 | 171 | 85 |
| *Trigona corvina* Cockerell, 1913 (TC) | 423 | 142 | 234 | 380 | 172 |
| *Trigona fulviventris* Guérin-Méneville, 1845 (TF) | 1298 | 242 | 168 | 387 | 535 |
| *Trigona fuscipennis* Friese, 1900 (TU) | 271 | 29 | 30 | 13 | 0 |
| *Trigona silvestriana* Vachal, 1908 (TS) | 27 | 126 | 12 | 28 | 1 |
| *Trigonisca buyssoni* Friese, 1902 (TB) | 3 | 34 | 89 | 15 | 7 |

| Scientific Name | Pan Trap | Vanr Trap | Aerial Net | Honey Spray | 30-minute observations | Total |
| --- | --- | --- | --- | --- | --- | --- |
| *Apis mellifera* Linneaus, 1758 (AM) | 22 | 2 | 242 | 659 | 428 | 1353 |
| *Cephalotrigona zexmeniae* Cockerell, 1912 (CZ) | 1 |  | 2 |  | 58 | 61 |
| *Ceratina auriviridis* H. S. Smith, 1907 (CA) |  | 1 | 1 |  | 3 | 5 |
| *Ceratina buscki* Cockerell, 1919 (CB) | 9 | 1 | 7 | 3 | 28 | 48 |
| *Ceratina chloris* Fabricius, 1804 (CH) |  |  |  |  | 1 | 1 |
| *Ceratina cobaltina* Cresson, 1878 (CC) | 3 | 1 | 6 |  | 15 | 25 |
| *Ceratina dimidiata* Friese, 1910 (CD) | 3 | 2 | 4 |  | 9 | 18 |
| *Ceratina eximia* Smith, 1862 (CE) | 15 | 2 | 24 | 2 | 35 | 78 |
| *Ceratina ignara* Cresson, 1878 (CI) | 5 |  | 19 | 3 | 2 | 29 |
| *Ceratina rectangulifera* Schwarz and Michener, 1954 (CR) | 4635 | 8 | 103 | 42 | 160 | 4948 |
| *Ceratina trimaculata* Friese, 1917 (CT) | 698 | 3 | 14 | 2 | 10 | 727 |
| *Ceratina zeteki* Cockerell, 1934 (CK) |  | 1 |  |  |  | 1 |
| *Lestrimelitta mourei* F. F. Oliveira and Marchi, 2005 (LM) |  |  | 1 |  |  | 1 |
| *Melipona beecheii* Bennett, 1831 (MB) | 1 |  | 2 | 3 | 1 | 7 |
| *Melipona fallax* Camargo and Pedro, 2008 (MF) |  |  | 10 | 2 | 25 | 37 |
| *Melipona costaricensis* Cockerell, 1919 (MA) | 2 |  | 20 | 2 | 8 | 32 |
| *Nannotrigona mellaria* Smith, 1862 (NM) |  |  | 5 | 6 | 26 | 37 |
| *Oxytrigona isthmina* Gonzalez and Roubik, 2008 (OI) | 1 |  | 10 |  |  | 11 |
| *Partamona orizabaensis* Strand, 1919 (PO) | 3 | 4 | 143 | 592 | 234 | 976 |
| *Plebeia frontalis* Friese, 1911 (PF) | 3 | 1 | 30 | 182 | 48 | 264 |
| *Plebeia pulchra* Ayala, 1999 (PP) | 7 | 4 | 83 | 412 | 94 | 600 |
| *Scaptotrigona mexicana* Guérin-Méneville, 1845 (SM) | 11 | 1 | 63 | 440 | 53 | 568 |
| *Scaptotrigona pectoralis* Dalla Torre, 1896 (SP) |  |  |  | 19 | 1 | 20 |
| *Tetragona dorsalis* Smith, 1854 (TD) |  |  | 27 | 19 | 118 | 164 |
| *Tetragonisca angustula* Latreille, 1811 (TA) | 7 |  | 114 | 547 | 201 | 869 |
| *Trigona corvina* Cockerell, 1913 (TC) | 3 | 1 | 109 | 576 | 239 | 928 |
| *Trigona fulviventris* Guérin-Méneville, 1845 (TF) | 7 | 7 | 304 | 66 | 948 | 1332 |
| *Trigona fuscipennis* Friese, 1900 (TU) | 1 | 1 | 37 | 10 | 23 | 72 |
| *Trigona silvestriana* Vachal, 1908 (TS) |  |  | 35 | 48 | 84 | 167 |
| *Trigonisca buyssoni* Friese, 1902 (TB) | 7 | 2 | 23 | 88 | 25 | 145 |
| **Total** | **5444** | **42** | **1438** | **3723** | **2877** | **13524** |

Supplementary Table S2. Full list of bee species in Tribes Apini, Ceratinini, and Meliponini surveyed in the San Luis Valley and the total number of bees collected per species from each sampling method

.

Supplementary Table S3**.** Results of LRT and lmerTest for abundance data collected from pan traps, vane traps and aerial netting samples combined for the 17 bee species statistically tested from these collection methods. Samples were collected along sampling paths located at 800m, 900m, 1000m, and 1100m elevation and along three replicate elevational gradients. When only the LRT is reported, the abundance was modeled with a negative binomial error distribution.

| *Bee Species* | *Likelihood Ratio Test* | *lmerTest* |
| --- | --- | --- |
| *Apis mellifera* Linneaus, 1758 | Chisq=2.50; p=0.48 | F_3,44_=0.30; p=0.82 |
| *Ceratina eximia* Smith, 1862 | Chisq=0.35; p=0.95 |  |
| *Ceratina ignara* Cresson, 1878 | Chisq=8.94; p=0.03 |  |
| *Ceratina rectangulifera* Schwarz and Michener, 1954 | Chisq=10.28; p=0.016 | F_3,42_=3.59; p=0.021 |
| *Ceratina trimaculata* Friese, 1917 | Chisq=7.92; p=0.005 | F_3,42_=4.12; p=0.012 |
| *Melipona costaricensis* Cockerell, 1919 | Chisq=8.94; p=0.03 |  |
| *Partamona orizabaensis* Strand, 1919 | Chisq=6.65; p=0.08 |  |
| *Plebeia frontalis* Friese, 1911 | Chisq=9.43; p=0.024 |  |
| *Plebeia pulchra* Ayala, 1999 | Chisq=2.45; p=0.485 |  |
| *Scaptotrigona mexicana* Guérin-Méneville, 1845 | Chisq=3.29; p=0.35 |  |
| *Tetragona dorsalis* Smith, 1854 | Chisq=11.895; p=0.008 |  |
| *Tetragonisca angustula* Latreille, 1811 | Chisq=0.275; p=0.96 |  |
| *Trigona corvina* Cockerell, 1913 | Chisq=3.10; p=0.38 |  |
| *Trigona fulviventris* Guérin-Méneville, 1845 | Chisq=7.41; p=0.009 |  |
| *Trigona fuscipennis* Friese, 1900 | Chisq=11.11; p=0.01 |  |
| *Trigona silvestriana* Vachal, 1908 | Chisq=10.78; p=0.002 |  |
| *Trigonisca buyssoni* Friese, 1902 | Chisq=3.38; p=0.34 |  |

Supplementary Table S4. Proportion of honey spray samples (n=116) containing each Meliponini bee species from each elevation during 2018-2019. Species present <5 samples were excluded from statistical analyses and are in bold. Species found to statistically differ by elevation are starred. Elevations with shared letters as superscripts do not statistically differ.

| Species | 800m | 900m | 1000m | 1100m | Total Proportion | χ^2^ | Pr (>χ^2^) |
| --- | --- | --- | --- | --- | --- | --- | --- |
| **Melipona beecheii Bennett, 1831 (MB)** | 0.000 | 0.000 | 0.029 | 0.000 | 0.009 |  |  |
| **Melipona fallax Camargo and Pedro, 2008 (MF)** | 0.000 | 0.000 | 0.029 | 0.036 | 0.017 |  |  |
| **Melipona costaricensis Cockerell, 1919 (MA)** | 0.042 | 0.033 | 0.000 | 0.000 | 0.017 |  |  |
| **Nannotrigona mellaria Smith, 1862 (NM)** | 0.083 | 0.067 | 0.000 | 0.000 | 0.034 |  |  |
| Partamona orizabaensis Strand, 1919 (PO) | 0.375 | 0.133 | 0.176 | 0.321 | 0.241 | 0.16 | 0.69 |
| Plebeia frontalis Friese, 1911 (PF)* | 0.208^b^ | 0.633^a^ | 0.088^b^ | 0.036^b^ | 0.241 | 9.77 | 0.001 |
| Plebeia pulchra Ayala, 1999 (PP) | 0.250 | 0.500 | 0.412 | 0.250 | 0.362 | 6.19 | 0.103 |
| Scaptotrigona mexicana Guérin-Méneville, 1845 (SM) | 0.083 | 0.167 | 0.118 | 0.214 | 0.147 | 1.73 | 0.189 |
| **Scaptotrigona pectoralis Dalla Torre, 1896 (SP)** | 0.042 | 0.067 | 0.000 | 0.036 | 0.034 |  |  |
| Tetragona dorsalis Smith, 1854 (TD)* | 0.167^a^ | 0.100^a^ | 0.000^b^ | 0.036^a^ | 0.069 | 4.81 | 0.028 |
| Tetragonisca angustula Latreille, 1811 (TA)* | 0.625^a^ | 0.467^ab^ | 0.500^a^ | 0.214^b^ | 0.448 | 5.77 | 0.016 |
| Trigona corvina Cockerell, 1913 (TC) | 0.208 | 0.133 | 0.176 | 0.107 | 0.155 | 0.008 | 0.930 |
| Trigona fulviventris Guérin-Méneville, 1845 (TF)* | 0.458^a^ | 0.167^ab^ | 0.118^b^ | 0.143^b^ | 0.207 | 5.25 | 0.021 |
| **Trigona fuscipennis** **Friese, 1900 (TU)** | 0.042 | 0.133 | 0.088 | 0.000 | 0.069 |  |  |
| Trigona silvestriana Vachal, 1908 (TS)* | 0.292^a^ | 0.000^b^ | 0.029^a^ | 0.000^b^ | 0.069 | 16.09 | <0.001 |
| Trigonisca buyssoni Friese, 1902 (TB) | 0.208 | 0.200 | 0.059 | 0.071 | 0.128 | 0.065 | 0.98 |

Supplementary Table S5. Proportion of timed flower observations (n=521) that each bee species was collected from each elevation during 2017-2019. Species with <10 individuals collected were excluded from statistical analyses and are in bold. Asterisks represent species found to statistically differ by elevation. Elevations with shared letters as superscripts do not statistically differ.

| Species | 800m | 900m | 1000m | 1100m | Total Proportion | *χ^2^* | *Pr (>χ^2^)* |
| --- | --- | --- | --- | --- | --- | --- | --- |
| *Apis mellifera* Linneaus, 1758 (AM) | 0.160 | 0.175 | 0.122 | 0.229 | 0.177 | 1.98 | 0.160 |
| *Ceratina buscki* Cockerell, 1919 (CB)* | 0.060^a^ | 0.026^a^ | 0.053^a^ | 0.006^b^ | 0.032 | 4.35 | 0.037 |
| *Ceratina cobaltina* Cresson, 1878 (CC) | 0.010 | 0.044 | 0.038 | 0.011 | 0.025 | 0.17 | 0.692 |
| ***Ceratina dimidiata* Friese, 1910 (CD)** | 0.010 | 0.018 | 0.015 | 0.011 | 0.013 |  |  |
| *Ceratina eximia* Smith, 1862 (CE) | 0.040 | 0.018 | 0.069 | 0.006 | 0.031 | 1.17 | 0.279 |
| *Ceratina rectangulifera* Schwarz and Michener, 1954 (CR)* | 0.210^a^ | 0.079^bc^ | 0.130^ab^ | 0.034^c^ | 0.101 | 16.32 | <0.001 |
| *Ceratina trimaculata* Friese, 1917 (CT)* | 0.000^b^ | 0.009^a^ | 0.023^a^ | 0.028^a^ | 0.017 | 4.14 | 0.042 |
| *Cephalotrigona zexmeniae* Cockerell, 1912 (CZ)* | 0.030^a^ | 0.026^a^ | 0.008^a^ | 0.000^b^ | 0.013 | 5.99 | 0.014 |
| ***Melipona beecheii* Bennett, 1831 (MB)** | 0.000 | 0.000 | 0.008 | 0.000 | 0.002 |  |  |
| *Melipona fallax* Camargo and Pedro, 2008 (MF)* | 0.000^b^ | 0.000^b^ | 0.008^a^ | 0.034^a^ | 0.013 | 9.85 | 0.002 |
| ***Melipona costaricensis* Cockerell, 1919 (MA)** | 0.000 | 0.026 | 0.000 | 0.011 | 0.010 |  |  |
| *Nannotrigona mellaria* Smith, 1862 (NM)* | 0.030^a^ | 0.026^a^ | 0.000^b^ | 0.000^b^ | 0.011 | 7.77 | 0.005 |
| *Partamona orizabaensis* Strand, 1919 (PO) | 0.140 | 0.167 | 0.069 | 0.229 | 0.158 | 2.99 | 0.083 |
| *Plebeia frontalis* Friese, 1911 (PF)* | 0.050^b^ | 0.158^a^ | 0.046^b^ | 0.000^ab^ | 0.055 | 12.08 | <0.001 |
| *Plebeia pulchra* Ayala, 1999 (PP)* | 0.130^a^ | 0.149^a^ | 0.137^a^ | 0.034^b^ | 0.103 | 8.63 | 0.003 |
| *Scaptotrigona mexicana* Guérin-Méneville, 1845 (SM) | 0.050 | 0.061 | 0.031 | 0.067 | 0.053 | 0.02 | 0.681 |
| ***Scaptotrigona pectoralis* Dalla Torre, 1896 (SP)** | 0.010 | 0.000 | 0.000 | 0.000 | 0.002 |  |  |
| *Tetragona dorsalis* Smith, 1854 (TD)* | 0.140^a^ | 0.061^ab^ | 0.153^a^ | 0.017^b^ | 0.084 | 8.27 | 0.004 |
| *Tetragonisca angustula* Latreille, 1811 (TA)* | 0.240^a^ | 0.272^a^ | 0.153^a^ | 0.028^b^ | 0.153 | 34.19 | <0.001 |
| *Trigona corvina* Cockerell, 1913 (TC)* | 0.160^a^ | 0.105^a^ | 0.084^ab^ | 0.017^b^ | 0.080 | 18.69 | <0.001 |
| *Trigona fulviventris* Guérin-Méneville, 1845 (TF)* | 0.230^b^ | 0.386^a^ | 0.496^a^ | 0.525^a^ | 0.431 | 29.69 | <0.001 |
| *Trigona fuscipennis* Friese, 1900 (TU)* | 0.060^a^ | 0.026^a^ | 0.015^a^ | 0.000^b^ | 0.021 | 11.15 | <0.001 |
| *Trigona silvestriana* Vachal, 1908 (TS)* | 0.190^a^ | 0.026^b^ | 0.038^b^ | 0.006^b^ | 0.053 | 33.75 | <0.001 |
| *Trigonisca buyssoni* Friese, 1902 (TB)* | 0.070^a^ | 0.044^a^ | 0.031^a^ | 0.000^b^ | 0.031 | 11.34 | <0.001 |

Supplementary Table S6. Number of individuals of each bee species collected near each elevation on *Persea Americana* (avocado) and *Cucurbita pepo* (squash) individuals in the San Luis Valley, Puntarenas province. An NA indicates that squash plants were not sampled at the 700m elevation band.

| Bee Species | Family | Crop | 700m | 800m | 900m | 1000m | 1100 m |
| --- | --- | --- | --- | --- | --- | --- | --- |
| *Apis mellifera* Linneaus, 1758 | Apidae | Avocado | 8 | 2 | 1 | 2 | 3 |
| *Habralictus* sp. 1 | Halictidae | Avocado | 0 | 0 | 0 | 2 | 2 |
| *Ceratina dimidiata* Friese, 1910 | Apidae | Avocado | 0 | 0 | 0 | 1 | 0 |
| *Ceratina eximia* Smith, 1862 | Apidae | Squash | NA | 0 | 1 | 2 | 0 |
| *Chilicola* sp. 1 | Apidae | Avocado | 0 | 0 | 0 | 0 | 2 |
| *Halictini* sp. 10 | Halictidae | Avocado | 0 | 0 | 0 | 2 | 3 |
| *Lasioglossum (Dialictus)* sp. 1 | Halictidae | Avocado | 0 | 1 | 0 | 0 | 0 |
| *Lasioglossum (Dialictus)* sp. 1 | Halictidae | Squash | NA | 1 | 4 | 0 | 7 |
| *Lasioglossum (Dialictus)* sp. 2 | Halictidae | Avocado | 0 | 0 | 0 | 0 | 1 |
| *Lasioglossum (Dialictus)* sp. 7 | Halictidae | Avocado | 0 | 0 | 0 | 0 | 1 |
| *Melissodes* sp. 1 | Apidae | Squash | NA | 1 | 2 | 10 | 15 |
| *Melitoma* sp. 1 | Apidae | Avocado | 0 | 0 | 1 | 1 | 0 |
| *Partamona orizabaensis* Strand, 1919 | Apidae | Avocado | 0 | 0 | 0 | 0 | 3 |
| *Partamona orizabaensis* Strand, 1919 | Apidae | Squash | NA | 0 | 3 | 0 | 10 |
| *Peponapis* sp. 4 | Apidae | Squash | NA | 0 | 1 | 1 | 4 |
| *Peponapis* sp. 5 | Apidae | Squash | NA | 0 | 0 | 1 | 0 |
| *Plebeia frontalis* Friese, 1911 | Apidae | Avocado | 2 | 0 | 2 | 0 | 0 |
| *Plebeia pulchra* Ayala, 1999 | Apidae | Avocado | 2 | 4 | 0 | 0 | 0 |
| *Scaptotrigona mexicana* Guérin-Méneville, 1845 | Apidae | Avocado | 2 | 8 | 6 | 4 | 14 |
| *Scaptotrigona pectoralis* Dalla Torre, 1896 | Apidae | Avocado | 1 | 0 | 0 | 0 | 0 |
| *Tetragona dorsalis* Smith, 1854 | Apidae | Avocado | 0 | 0 | 1 | 0 | 0 |
| *Tetragonisca angustula* Latreille, 1811 | Apidae | Avocado | 11 | 8 | 13 | 0 | 0 |
| *Thygater* sp. 4 | Apidae | Squash | NA | 3 | 0 | 0 | 1 |
| *Trigona corvina* Cockerell, 1913 | Apidae | Avocado | 1 | 1 | 8 | 1 | 0 |
| *Trigona fulviventris* Guérin-Méneville, 1845 | Apidae | Avocado | 0 | 0 | 0 | 1 | 5 |
| *Trigona fulviventris* Guérin-Méneville, 1845 | Apidae | Squash | NA | 0 | 6 | 96 | 14 |
| *Trigona fuscipennis* Friese, 1900 | Apidae | Avocado | 0 | 2 | 5 | 0 | 0 |
| *Trigona silvestriana* Vachal, 1908 | Apidae | Avocado | 0 | 3 | 0 | 0 | 0 |
| *Trigona silvestriana* Vachal, 1908 | Apidae | Squash | NA | 14 | 1 | 24 | 1 |
| *Trigonisca buyssoni* Friese, 1902 | Apidae | Avocado | 2 | 0 | 2 | 0 | 0 |

**APPENDIX S2-**Supplementary figures

Supplementary Figure S1. Rarefaction curves comparing bee species richness of Meliponine and Ceratinine bee species at four elevations. 95% CI bars reflect the variation among the replicate elevational gradients*.* An abundance matrix was used for the species richness estimates and species richness was rescaled by abundance on the x-axis. Bee abundance was totaled for that sampling path from all pan trap, vane trap, and aerial netting samples.


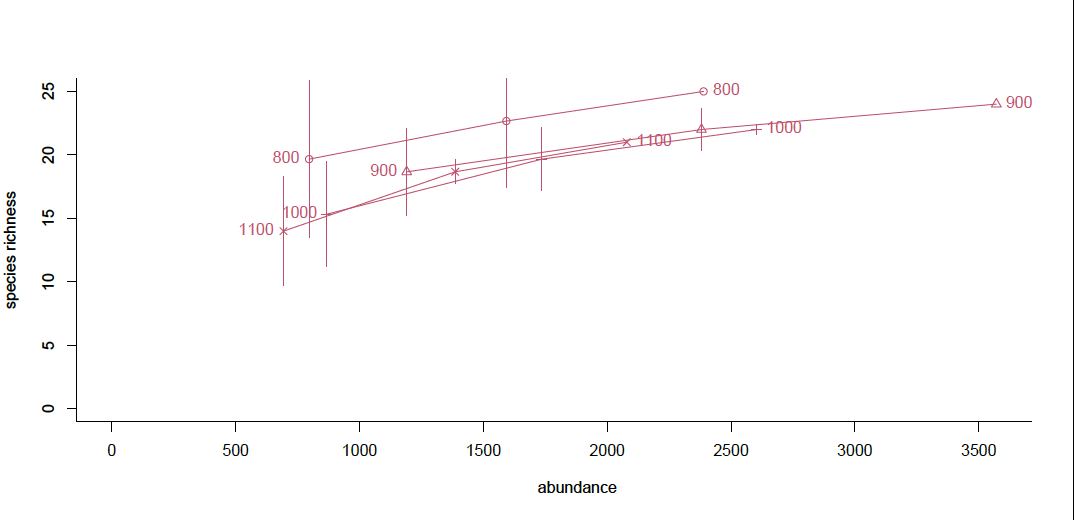


Supplementary Figure S2. Mean (± SE) abundance for 17 species of bees collected from pan traps, vane traps and aerial netting samples. Total abundance across the three sampling methods was summed by year for four years (2016, 2017, 2018 and 2019). Means (± SE) are the average of the four years and across the three replicate elevational gradients, comparing bee abundance for the four elevations. Letters show a statistically significant difference in abundance between elevations not sharing letters.


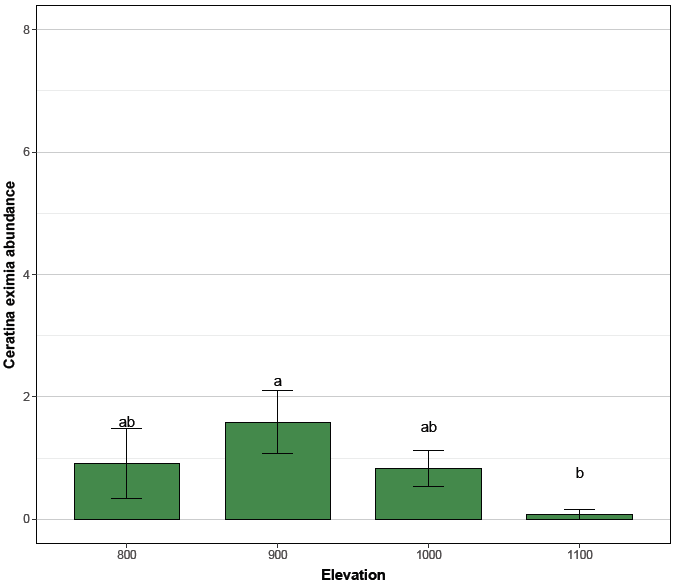


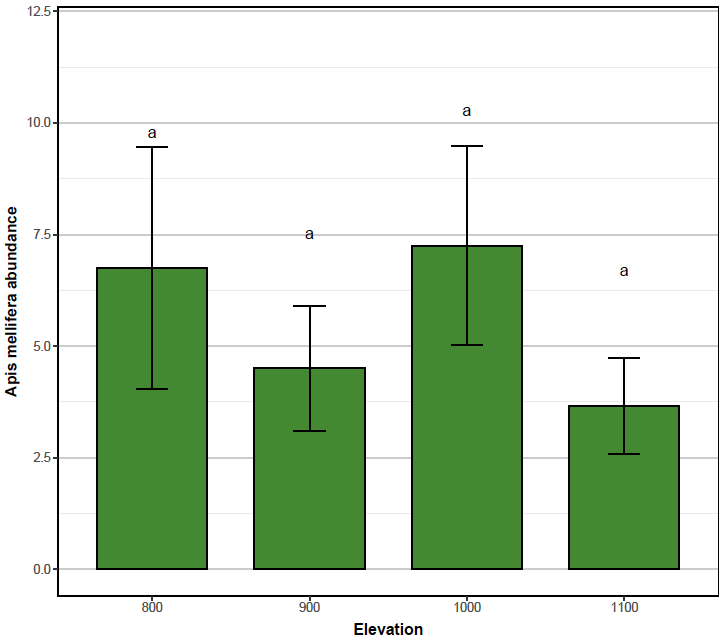


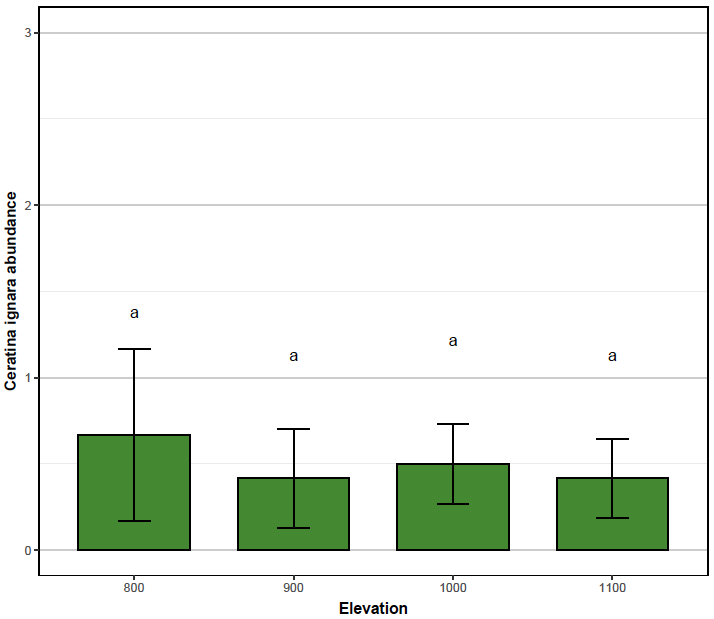

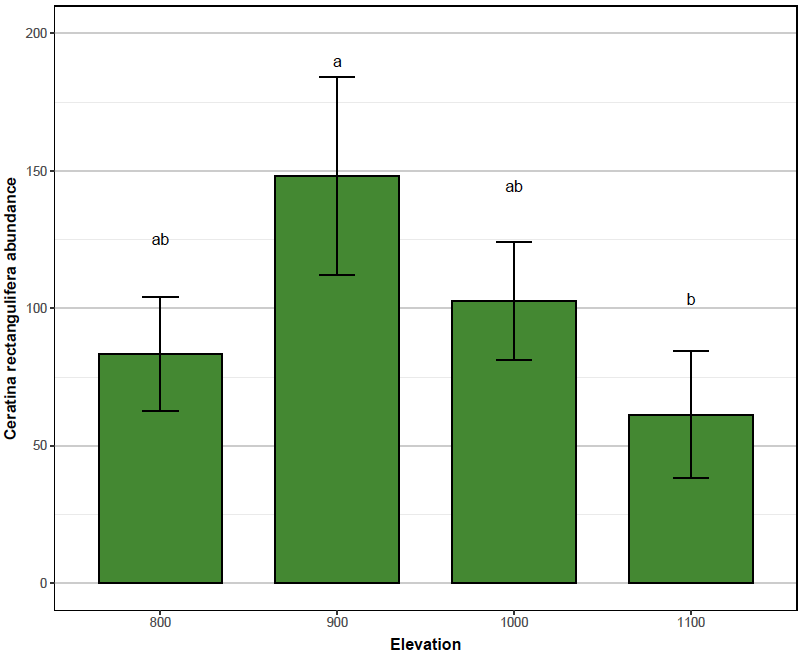


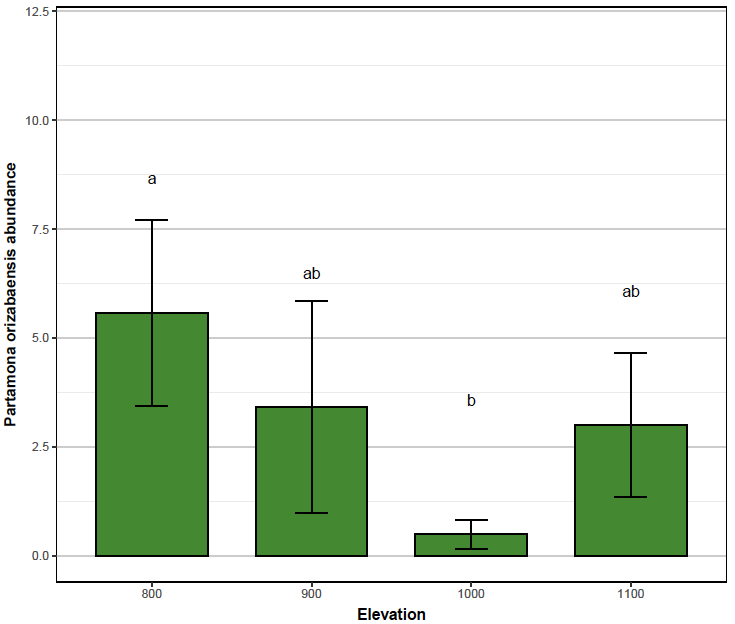

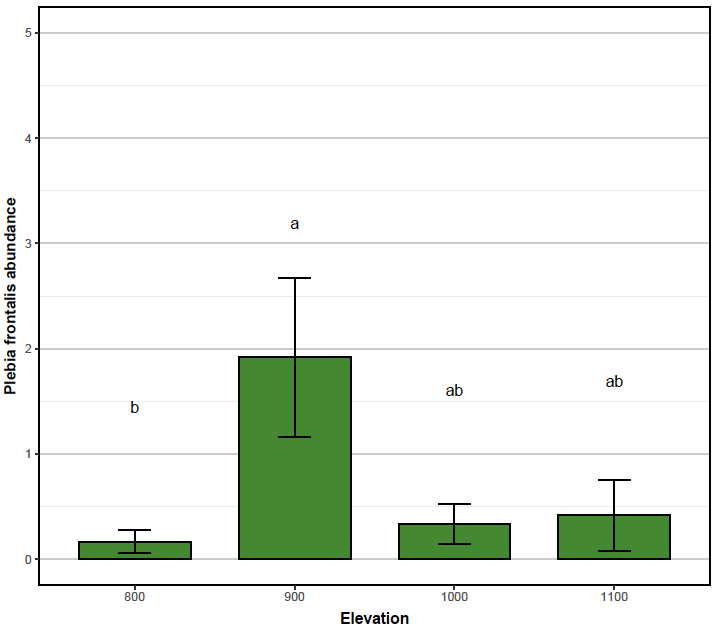

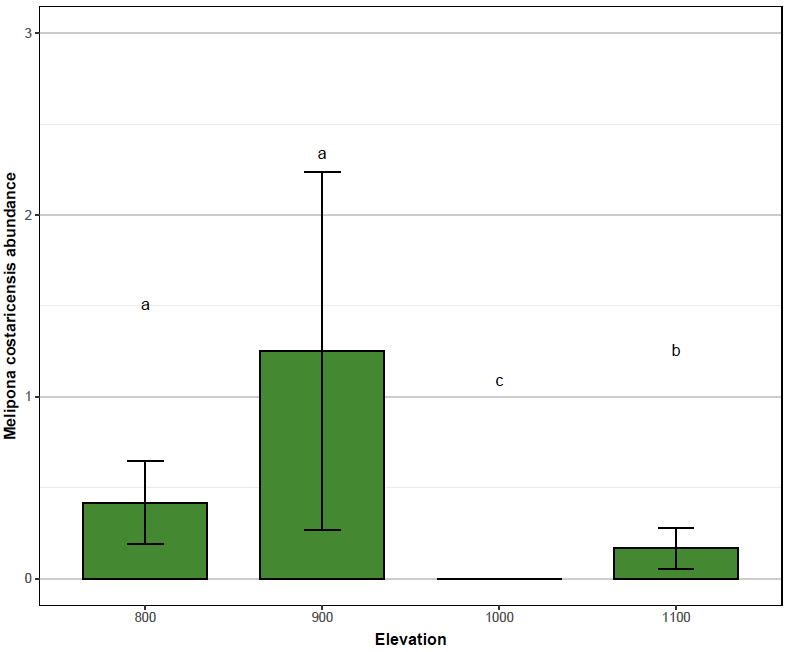

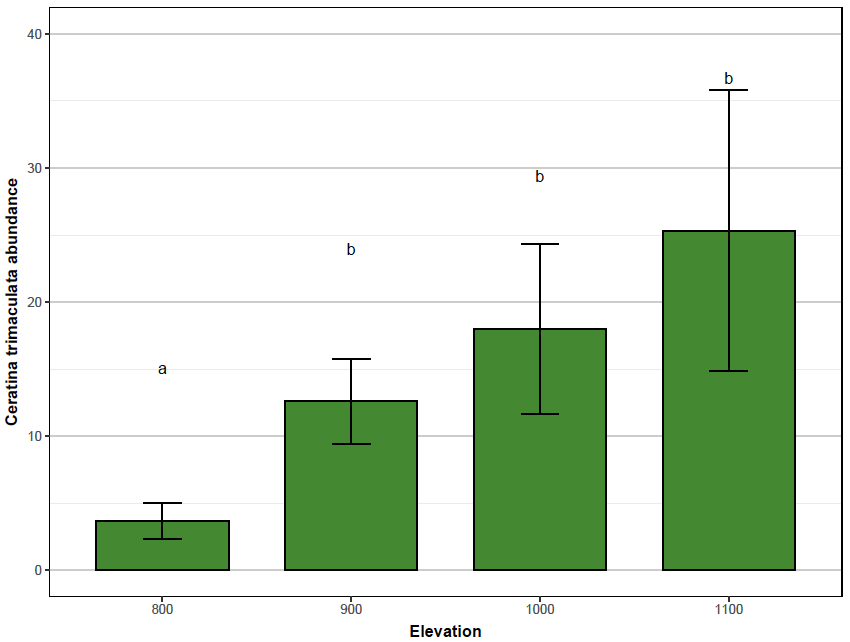


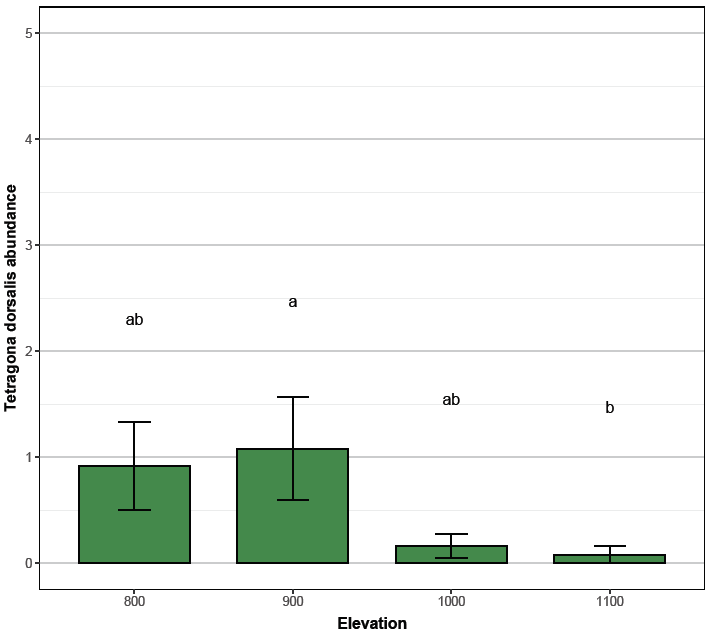

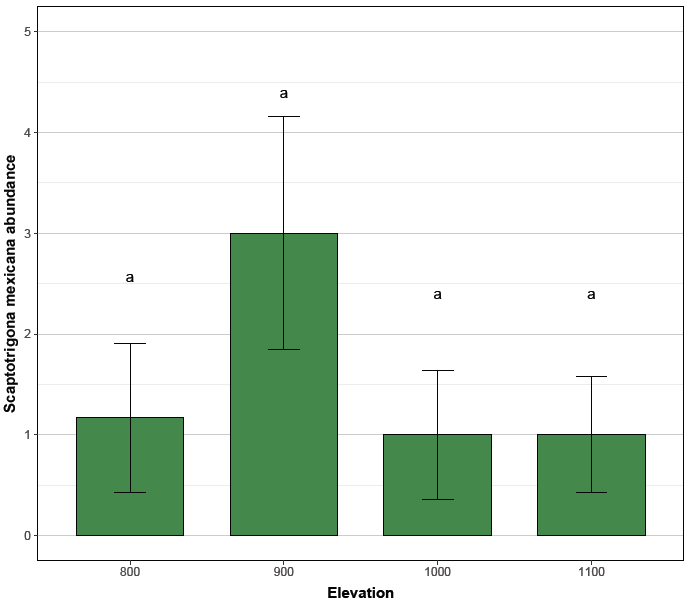

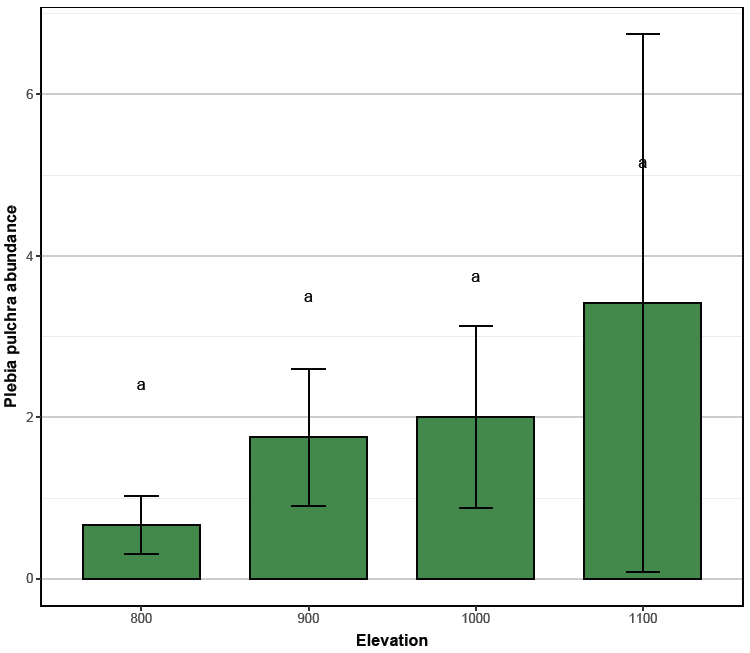


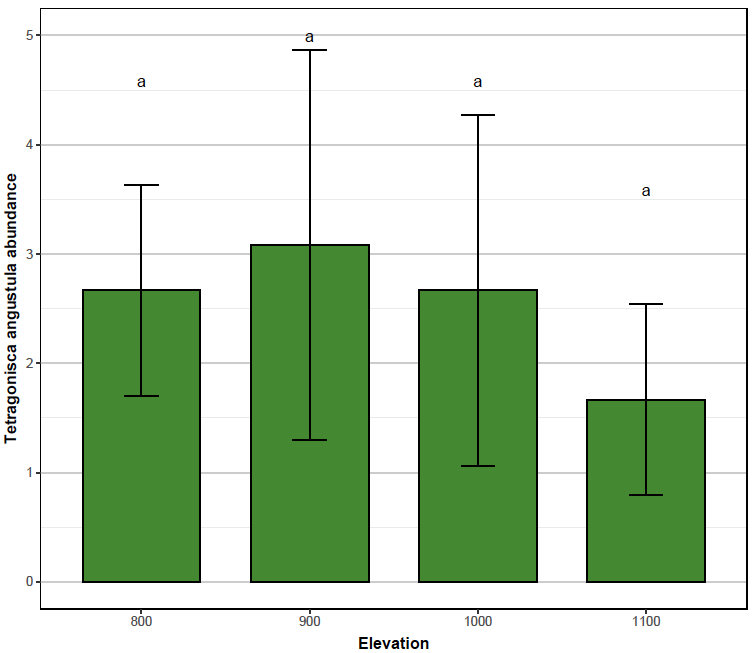


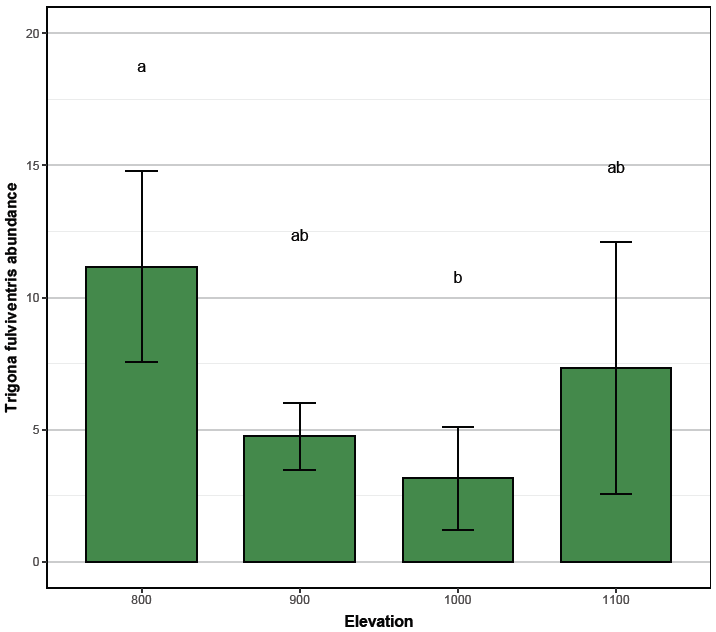


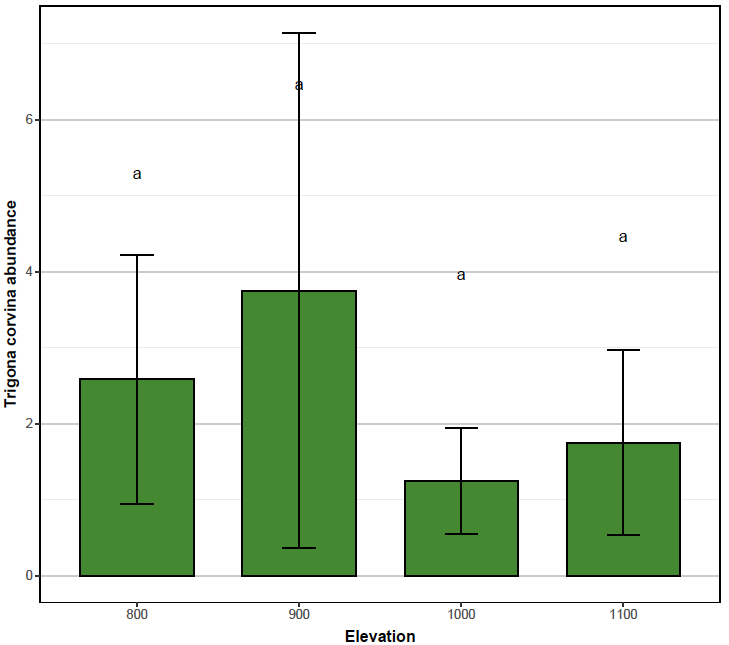


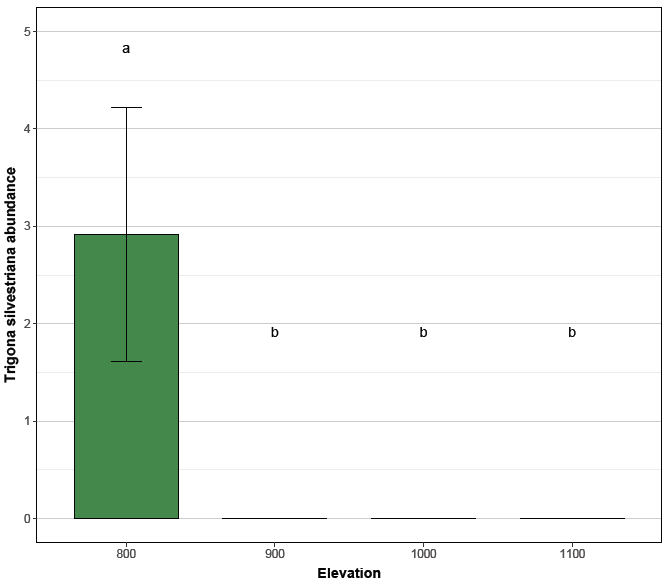

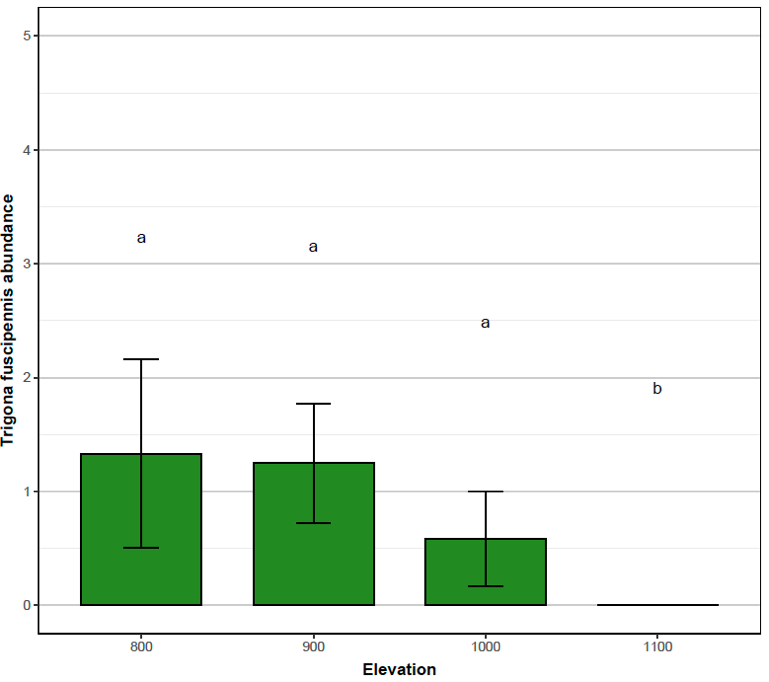


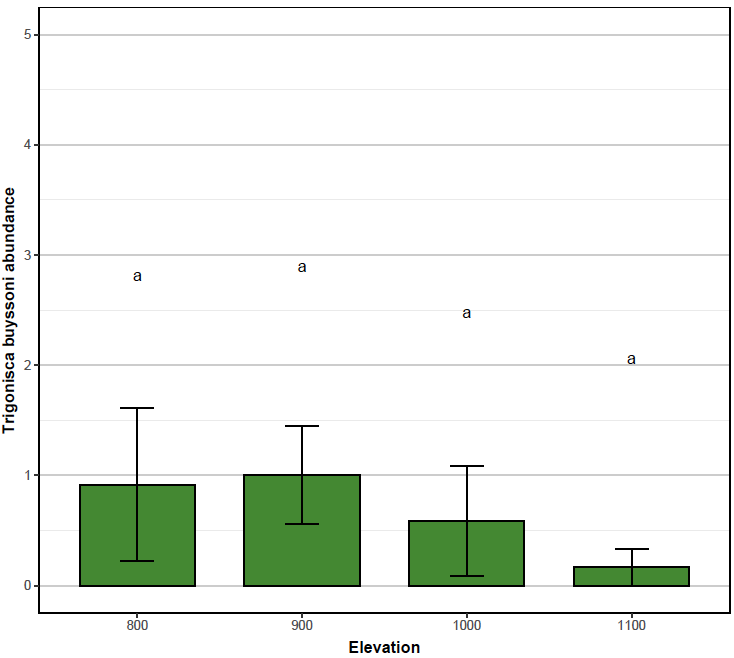


Supplementary Figure S3. Permutational Analysis of Similarity (ANOSIM) comparing bee community dissimilarity between wet season and dry season assemblages. Dissimilarity matrix was constructed using the Chao dissimilarity index. Permutation ran 999 iterations.


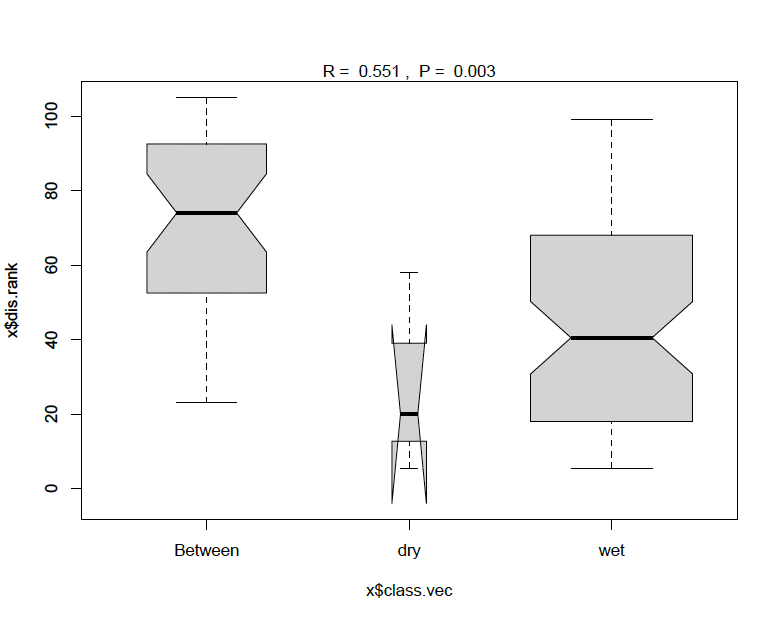


Supplementary Figure S4. Mean (± SE) abundance for 22 species of bees collected from pan traps, vane traps and aerial netting samples. Total abundance across the three sampling methods was summed along all elevations and replicate elevational gradients within a sampling period. June and July sampling periods were assigned to the wet season (n=11) and December sampling periods were assigned to the dry season (n=3). Means (± SE) are the average of the four years and across the three replicate elevational gradients, comparing bee abundance between the two seasons. Letters show a statistically significant difference in abundance between seasons not sharing letters.


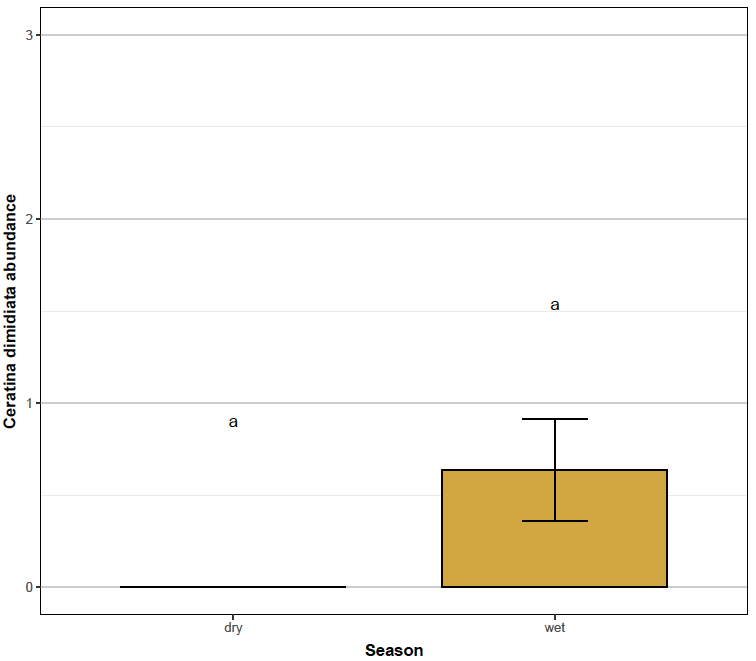

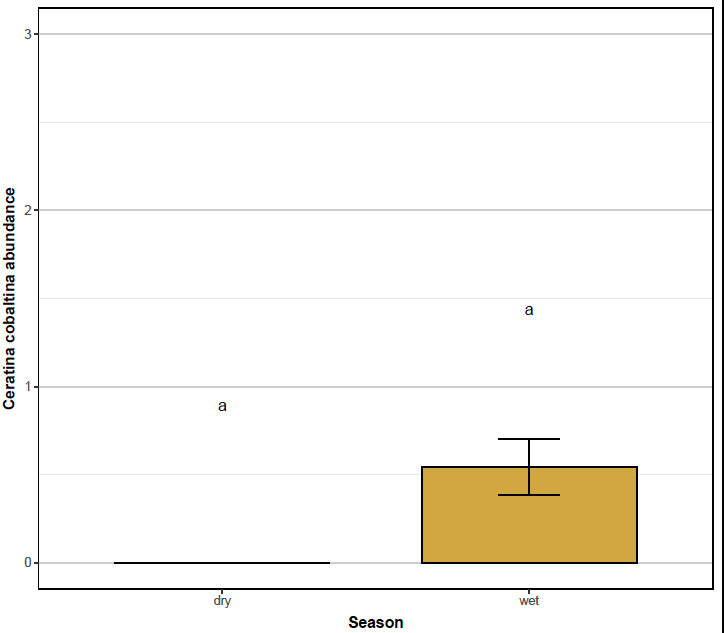

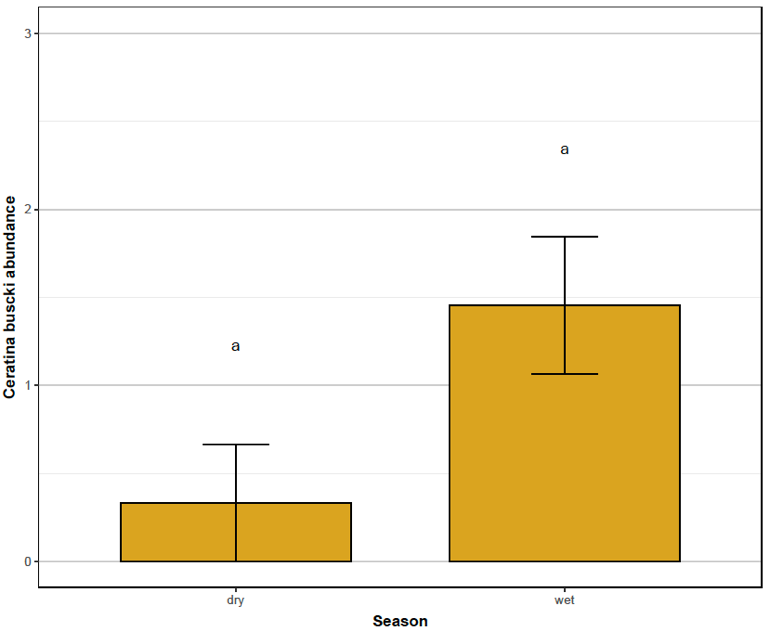

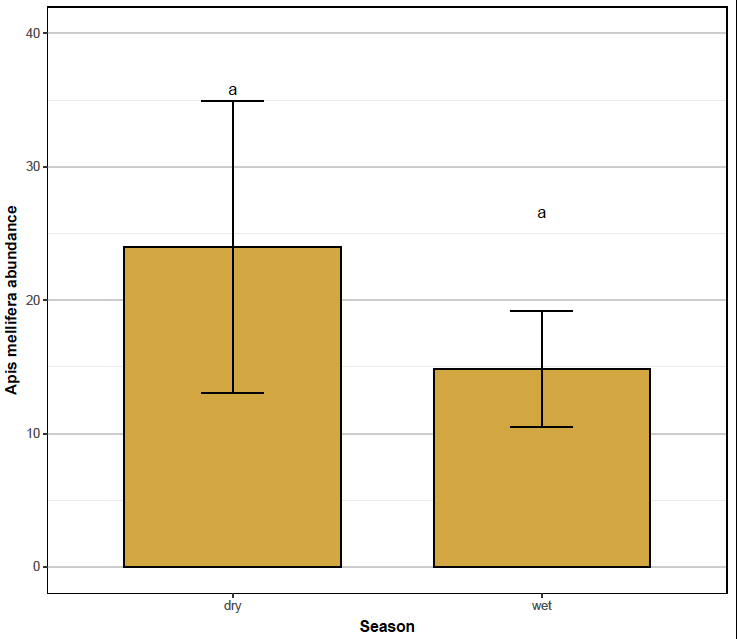


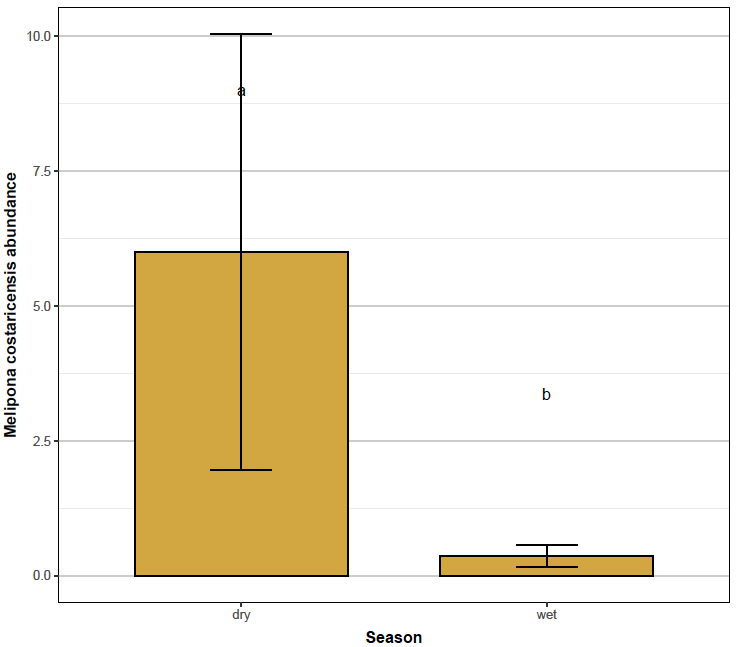

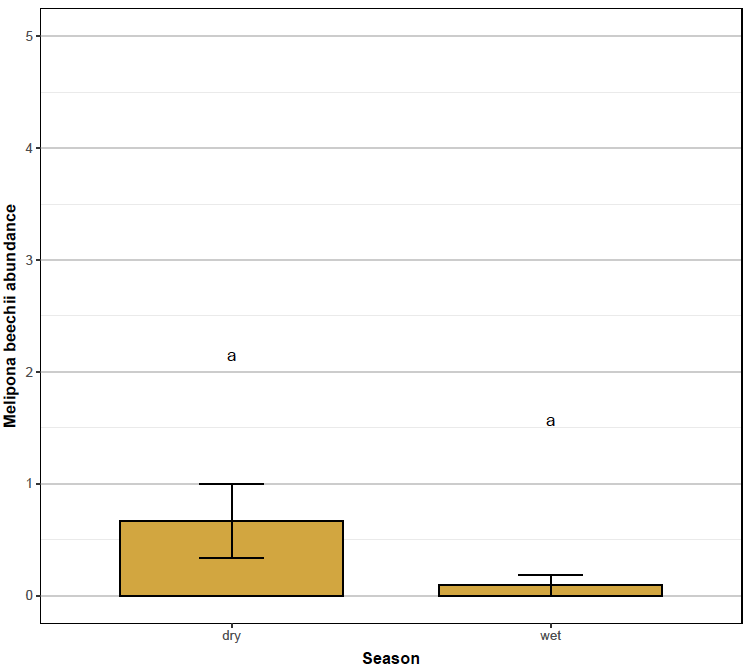

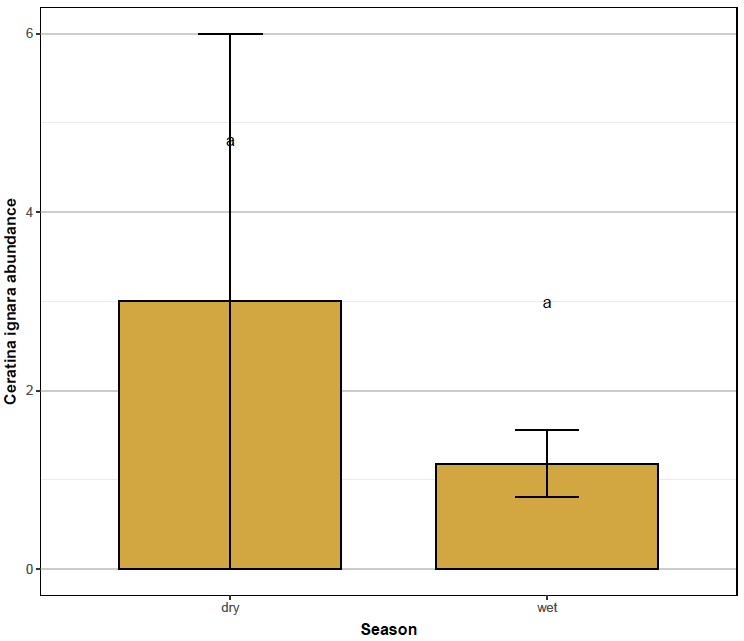

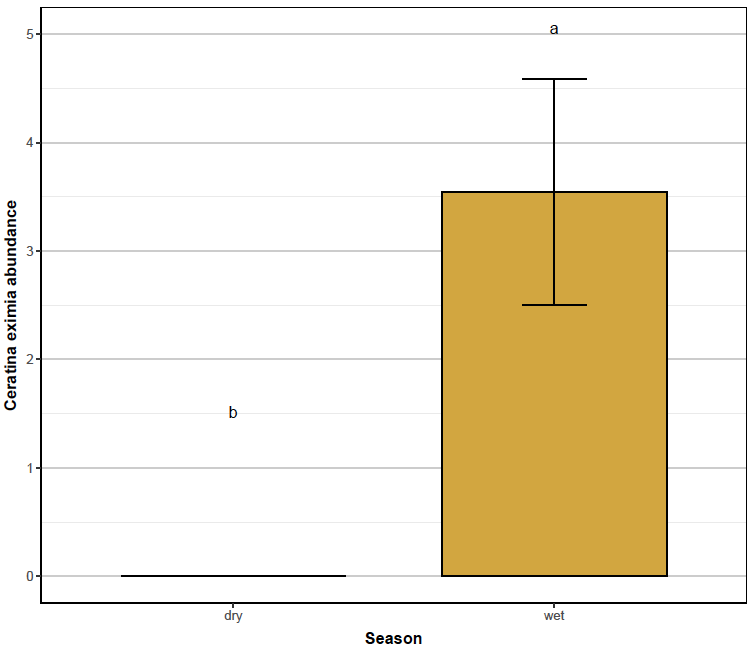


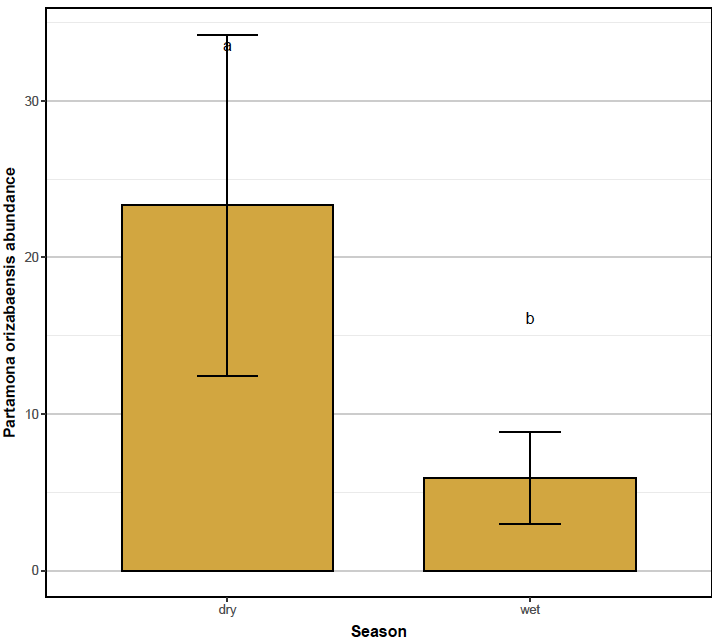


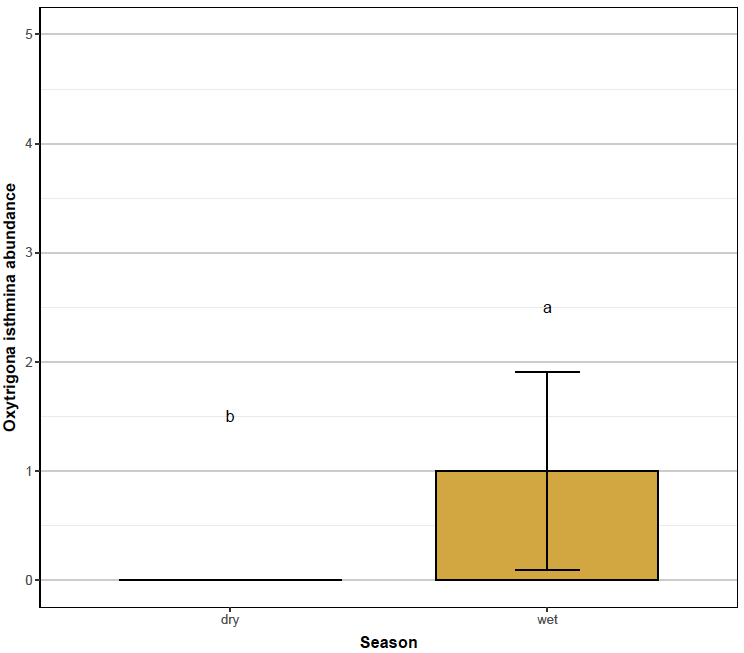

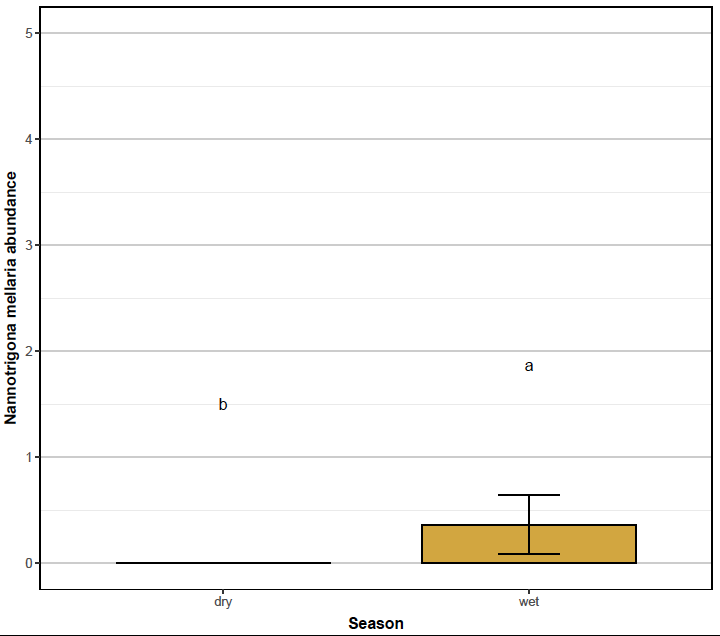

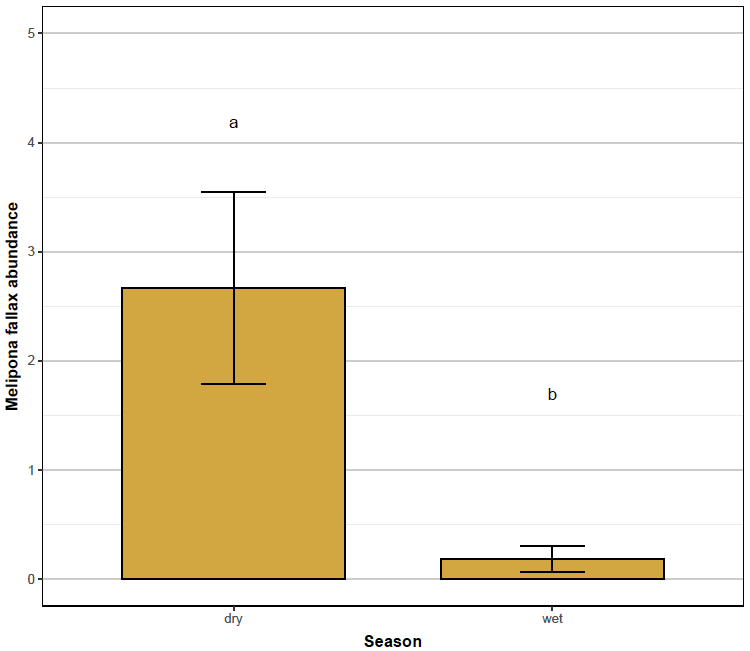


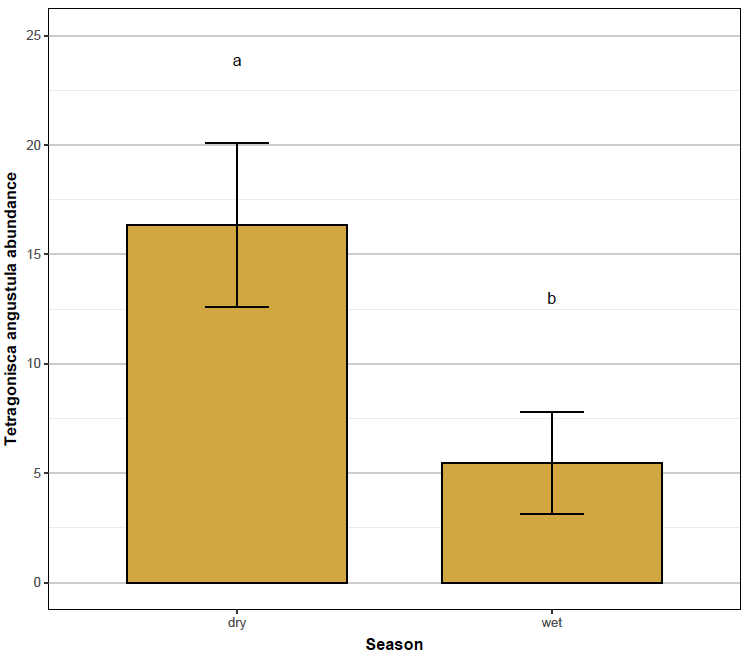

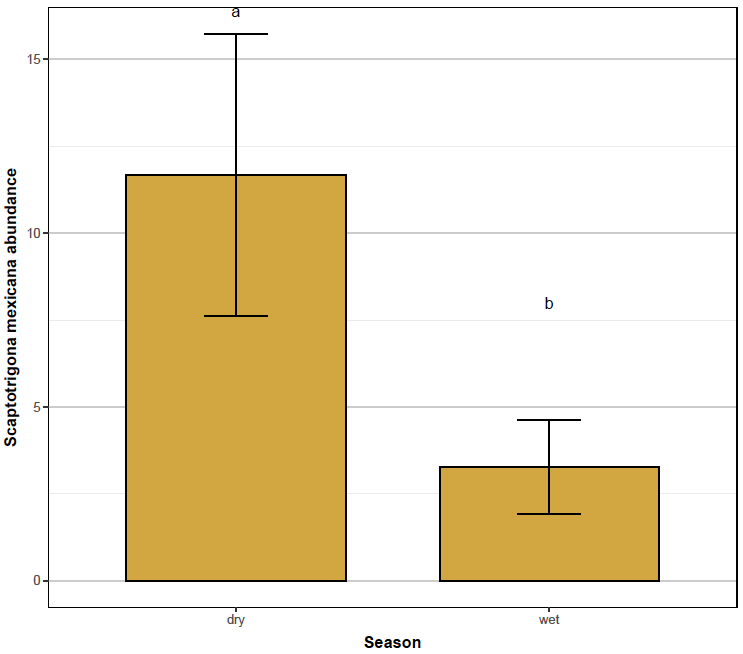

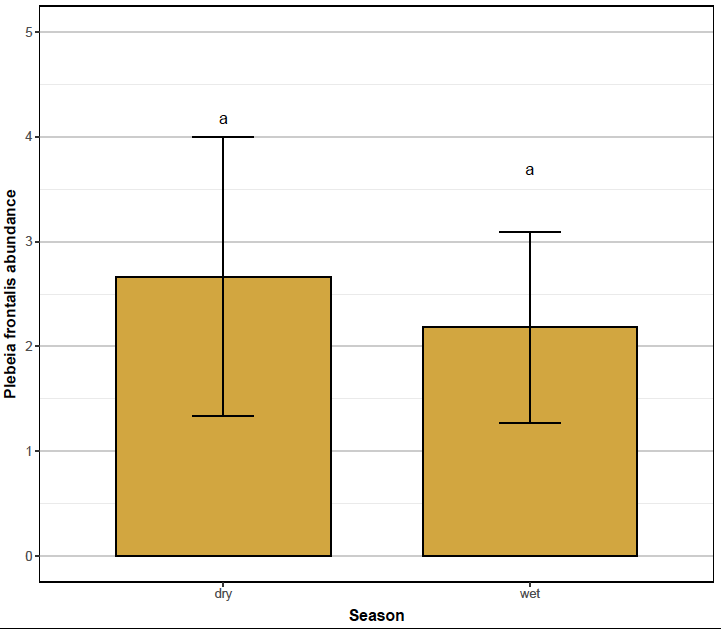

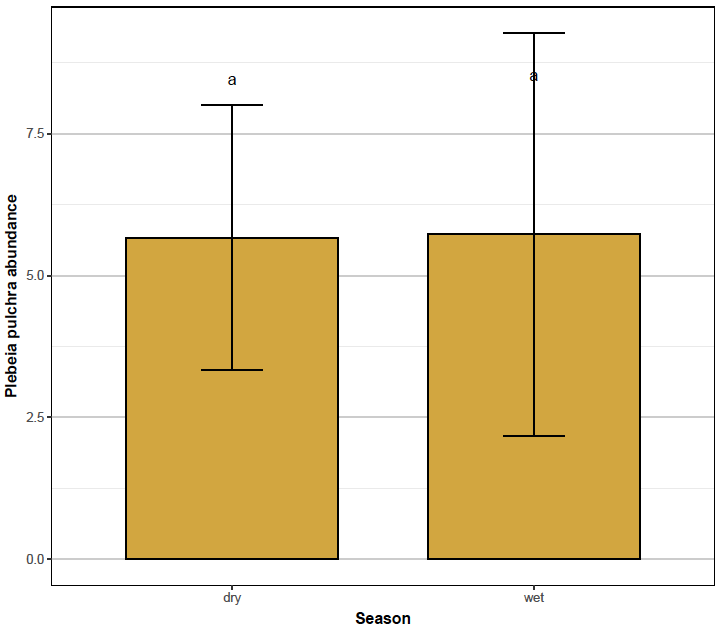


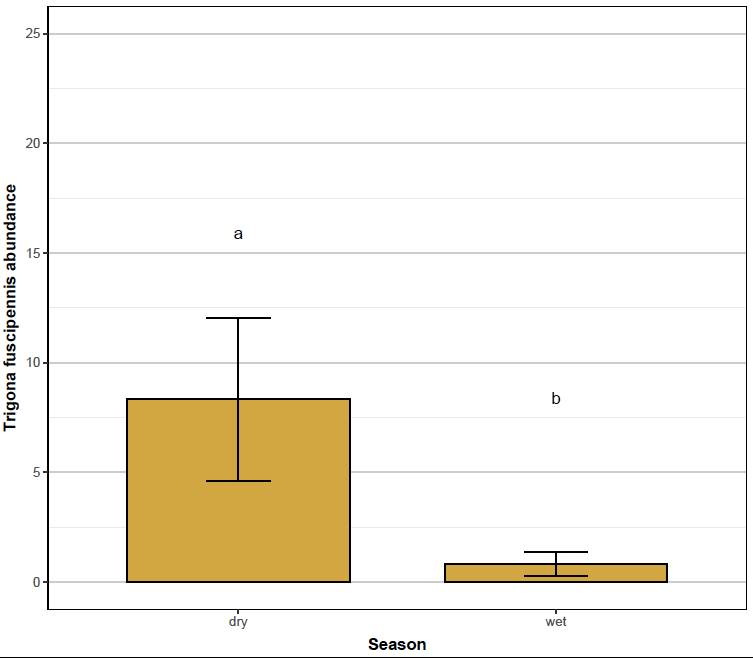

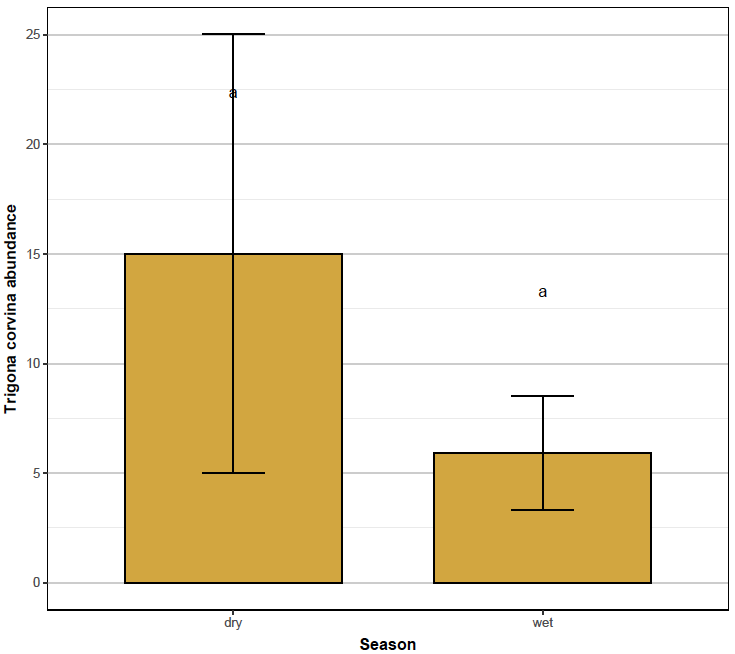

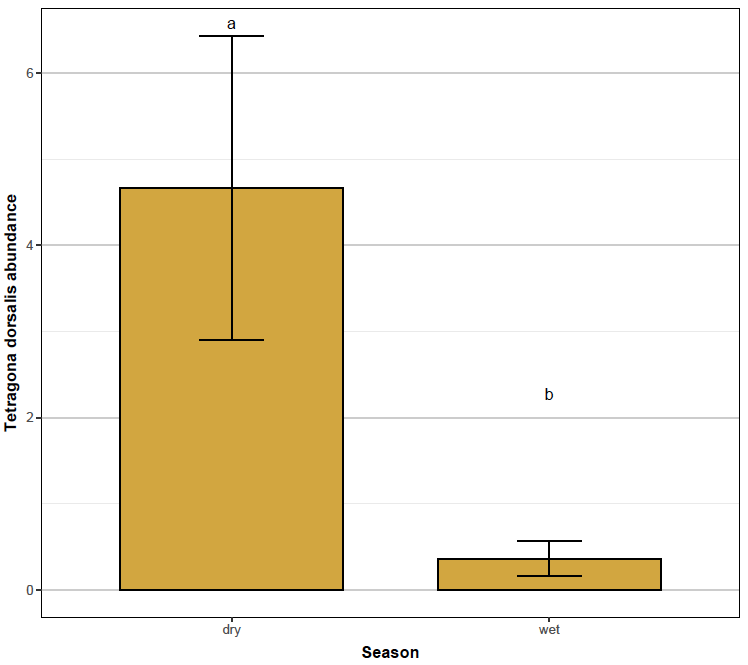


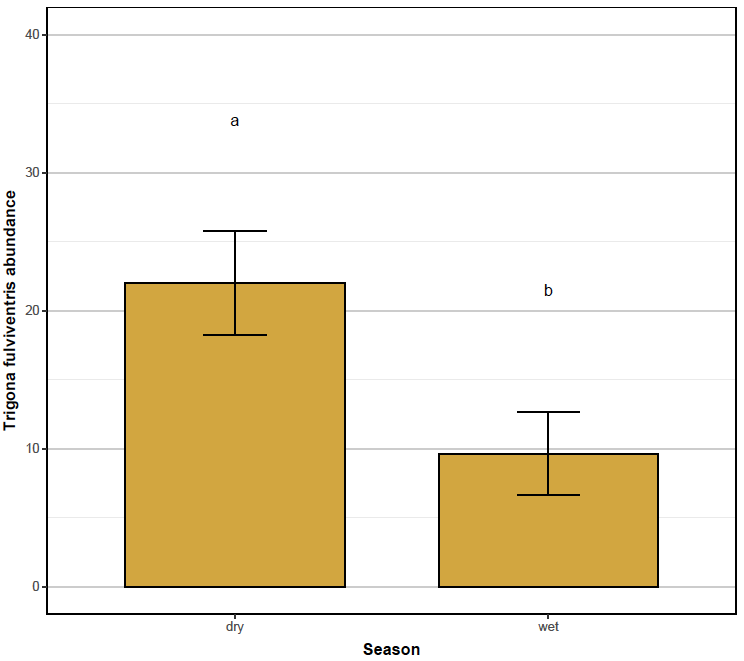


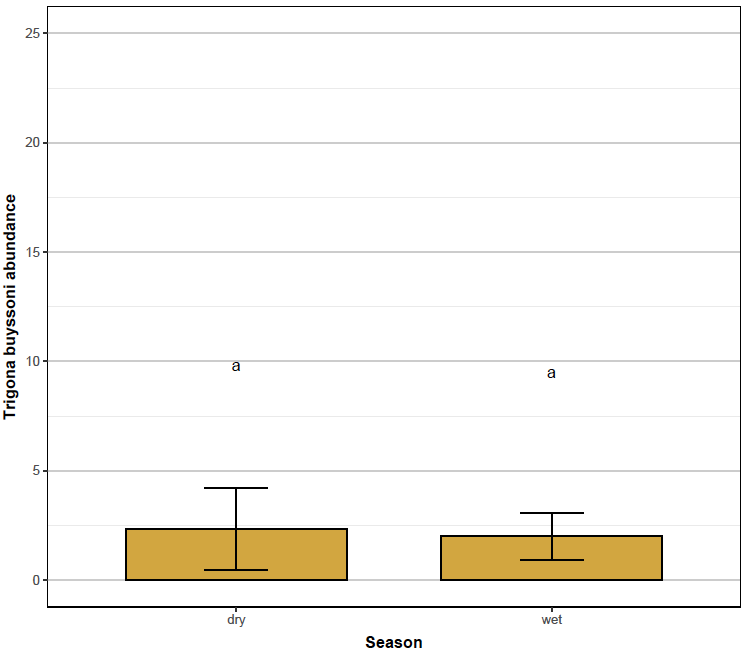

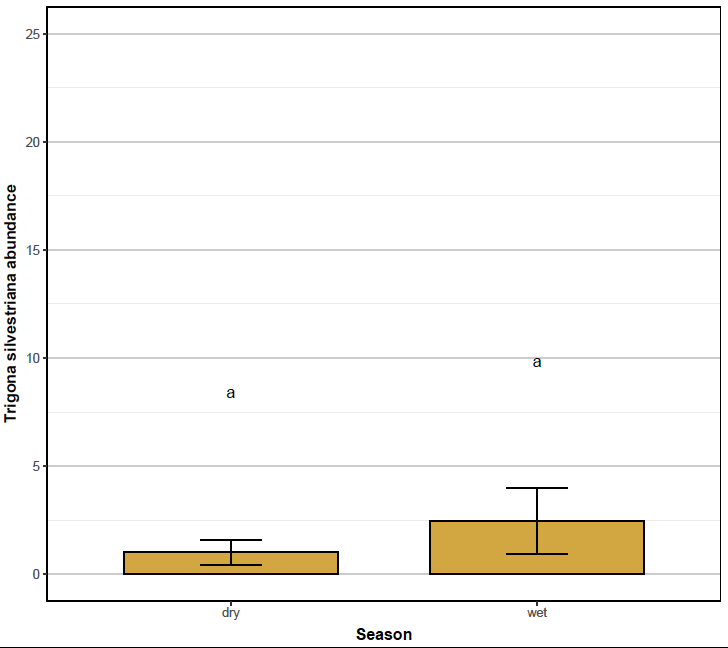


Supplementary Figure S5. A subset of 2,877 bees representing three tribes from a total of 3,868 bees that were collected directly from flowering plant species during 30-minute timed observations. Exact elevations, ranging from 700m elevation to 1150m elevation were used to create each boxplot, where the median elevation of collection is denoted by the median line in the center of each box. Red dots show the exact elevation that each individual was collected, with boxplot whiskers comprising the full range of detection of each species. An asterisk denotes a statistically significant effect of elevation on bee species presence using a binomial glm (p < 0.05). Each red dot is one individual.

Supplementary Figure S6. The three replicate elevational gradients located in San Luis de Monteverde, Costa Rica are delineated in red. Sampling path locations along each elevational gradient is shown by each black and white circle with the elevation of the sampling path labeled, for a total of 12 sampling paths. Avocado sampling locations are indicated by yellow pins, squash sampling locations are indicated by blue pins, and locations where both crop species were sampled are indicated by green pins. Obtained from Google Earth Pro version 7.3.3; [https://earth.google.com/web/](https://earth.google.com/web/@0,0,0a,22251752.77375655d,35y,0h,0t,0r).


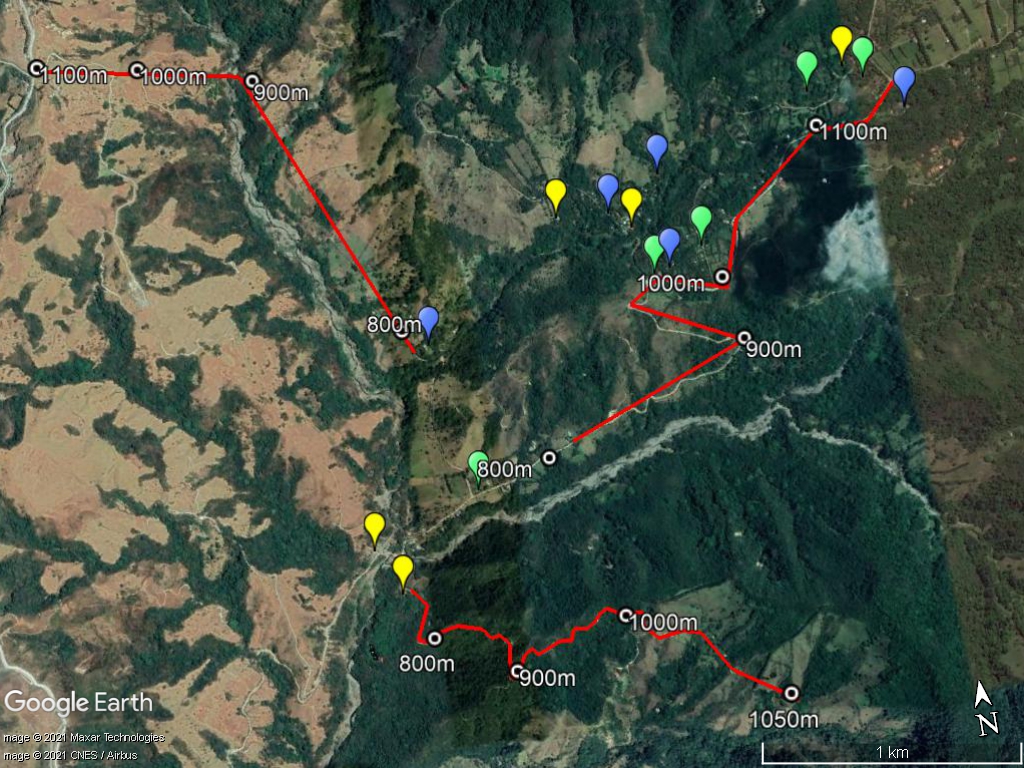


Supplementary Figure S7. Mean maximum and average monthly temperatures (°C) for (a) 800 m elevation and (b) 1100 m elevation in San Luis de Monteverde, CR, 1970-2000 and (c) the differences in the temperatures between 800 and 1100 m.a.s.l. in San Luis de Monteverde, CR, 1970-2000. Maps show the study area location within Costa Rica, and mean maximum and average temperatures for Costa Rica. Both maps show that the study area is located in the hottest and driest region of Costa Rica, the Pacific Northwest. Obtained using the ‘raster’ and ‘maps’ packages of R versions 3.1-5 and 3.3.0. <https://cran.r-project.org/web/packages/maps/index.html>; <https://cran.r-project.org/web/packages/raster/index.html>.

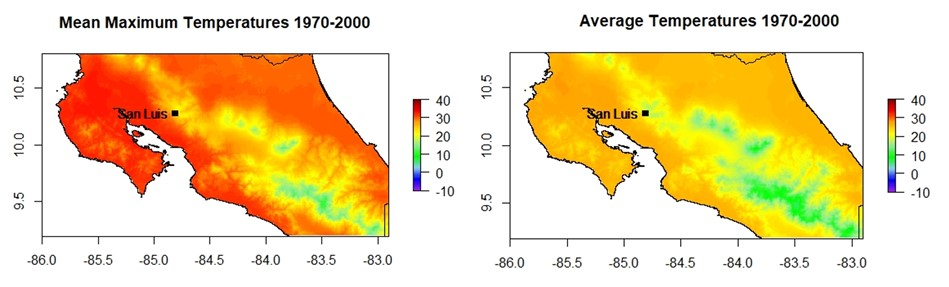


Supplementary Figure S8. Species accumulation curves for timed samples conducted at flowers of *Persea americana* and *Cucurbita pepo*, 2018-2019.


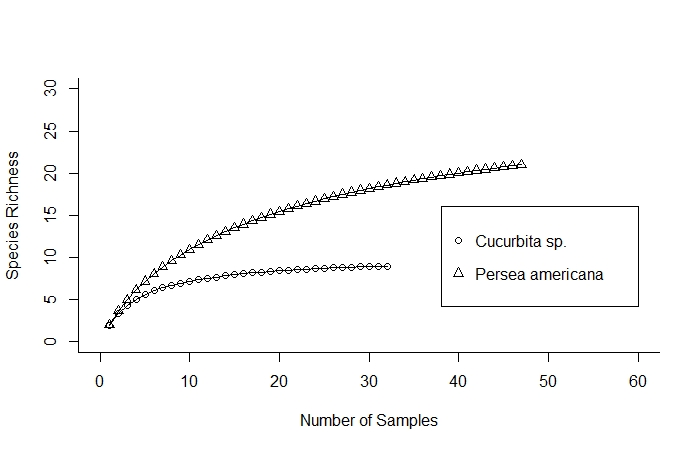


Supplementary Figure S9. Rarefaction curves for timed samples conducted at flowers of *Persea americana* and *Cucurbita pepo*, 2018-2019. The x-axis is rescaled to the number of individuals collected.


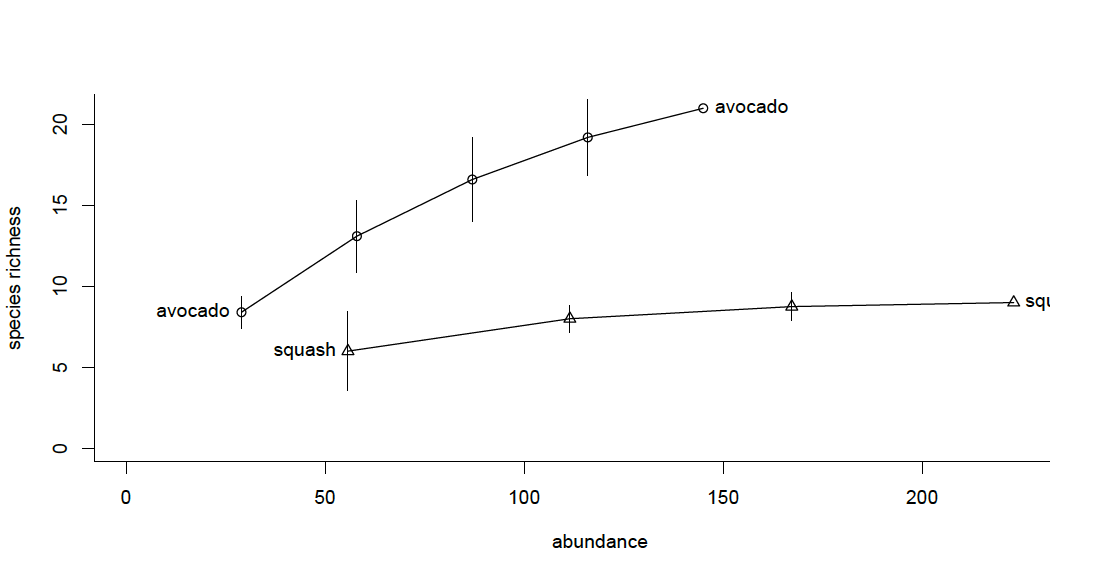


Supplementary Figure S10. Bee species relative abundance by elevation and crops species. Crop species explained 57% of the variation in bee community composition while elevation was significant in explaining 17% of the variation in bee community composition.


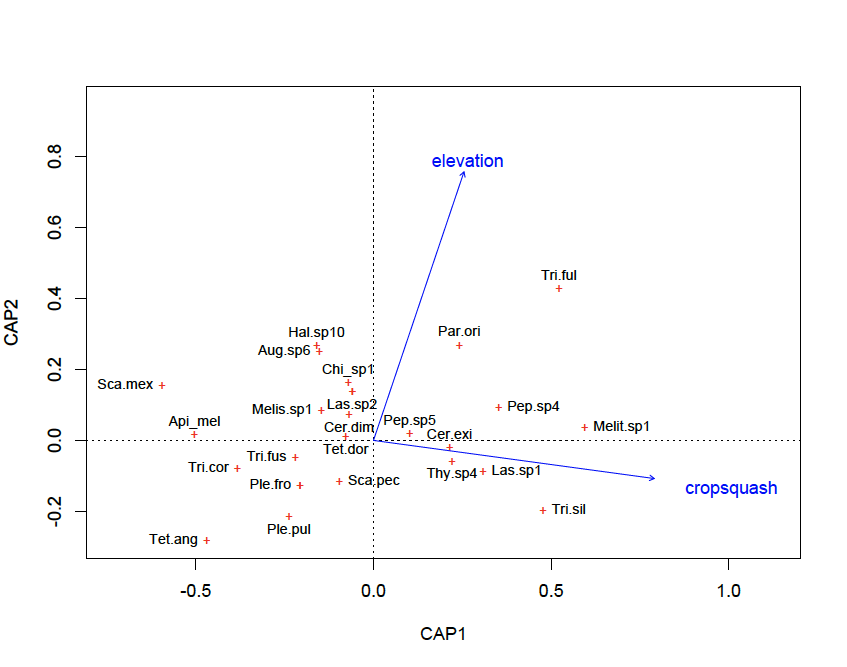


Supplementary Figure S11. Distance-decay relationship between spatial distances between sampling paths and bee community dissimilarity. Dissimilarities were constructed using the Bray-Curtis distance index. Spatial distances were calculated using each sampling paths UTM coordinates.


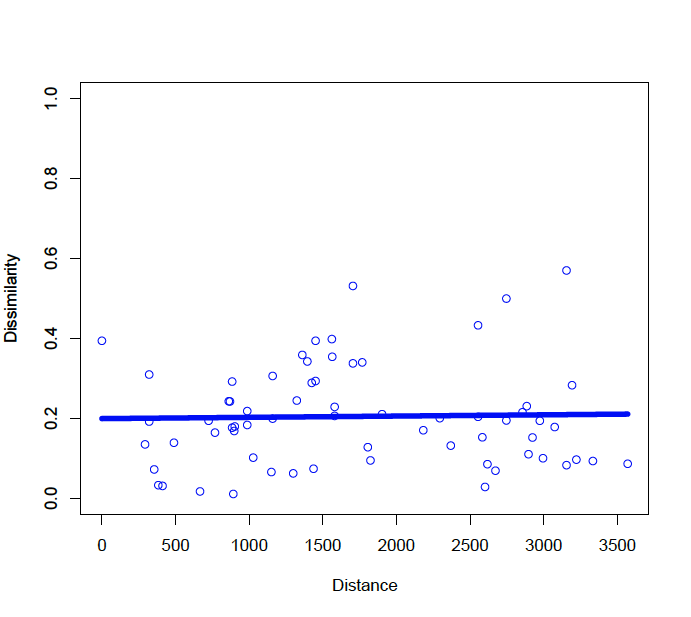


1. [↑](#footnote-ref-1)
